# Inner/Outer Side Chain Engineering of Non‐Fullerene Acceptors for Efficient Large‐Area Organic Solar Modules Based on Non‐Halogenated Solution Processing in Air

**DOI:** 10.1002/advs.202405716

**Published:** 2024-07-16

**Authors:** Sabeen Zahra, Seungjin Lee, Muhammad Jahankhan, Muhammad Haris, Du Hyeon Ryu, Bumjoon J. Kim, Chang Eun Song, Hang Ken Lee, Sang Kyu Lee, Won Suk Shin

**Affiliations:** ^1^ Advanced Energy Materials Research Center Korea Research Institute of Chemical Technology (KRICT) Daejeon 34114 Republic of Korea; ^2^ Advanced Materials and Chemical Engineering University of Science and Technology (UST) Daejeon 34113 Republic of Korea; ^3^ Department of Chemical and Biomolecular Engineering Korea Research Institute of Science and Technology (KAIST) Daejeon 34141 Republic of Korea

**Keywords:** large‐area organic solar modules, morphology control, non‐halogenated solvents, room temperature processing, Y6 alkyl‐chain modification

## Abstract

Achieving efficient and large‐area organic solar modules via non‐halogenated solution processing is vital for the commercialization yet challenging. The primary hurdle is the conservation of the ideal film‐formation kinetics and bulk‐heterojunction (BHJ) morphology of large‐area organic solar cells (OSCs). A cutting‐edge non‐fullerene acceptor (NFA), Y6, shows efficient power conversion efficiencies (PCEs) when processed with toxic halogenated solvents, but exhibits poor solubility in non‐halogenated solvents, resulting in suboptimal morphology. Therefore, in this study, the impact of modifying the inner and outer side‐chains of Y6 on OSC performance is investigated. The study reveals that blending a polymer donor, PM6, with one of the modified NFAs, namely N‐HD, achieved an impressive PCE of 18.3% on a small‐area OSC. This modified NFA displays improved solubility in *o*‐xylene at room temperature, which facilitated the formation of a favorable BHJ morphology. A large‐area (55 cm^2^) sub‐module delivered an impressive PCE of 12.2% based on N‐HD using *o*‐xylene under ambient conditions. These findings underscore the significant impact of the modified Y6 derivatives on structural arrangements and film processing over a large‐area module at room temperature. Consequently, these results are poised to deepen the comprehension of the scaling challenges encountered in OSCs and may contribute to their commercialization.

## Graphical abstract

Inner/outer side‐chain modification of non‐fullerene acceptors (NFAs) has a pronounced impact on large‐area film formation at room temperature. Herein, a highly efficient sub‐module is reported by designing a series of Y6 derivatives with modified branched inner/outer side chains. The optimized NFA blend exhibits the best power conversion efficiency of 12.2% with a 55 cm^2^ photoactive area.

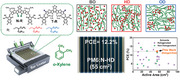

## 1 Introduction

Recently, bulk‐heterojunction (BHJ) organic solar cells (OSCs) have attracted much attention due to their lightweight properties, flexibility/stretchability, excellent solution processability, suitability in indoor applications, and semi‐transparency through high‐throughout printing techniques.^[^ ^1^, ^2^, ^3^, ^4^, ^5^ ^]^ In the past few years, OSCs have made significant progress in power conversion efficiencies (PCEs) due to the rapid development of non‐fullerene acceptors (NFAs).^[^ ^6^, ^7^, ^8^, ^9^ ^]^ In this regard, a state‐of‐the‐art NFA, namely Y6, and its derivatives have boosted their PCEs to over 19% and have shown great potential for device area scale‐up.^[^ ^10^, ^11^, ^12^, ^13^ ^]^

Nevertheless, current highly efficient OSCs encounter several challenges when transitioning device production from the laboratory to the factory. 1) Many high‐performance devices are predominantly fabricated using the spin‐coating technique. While spin‐coating allows for simple and precise control over film formation in small areas, its applicability to large‐area modules is hindered by drawbacks such as wasteful material usage and non‐uniform film thickness across large substrates. 2) The adoption of scalable‐printing methods (e.g., blade coating, slot‐die coating, and bar coating) presents an alternative to spin coating. However, these methods require meticulous control to achieve optimal device performance.^[^ ^14^ ^]^ It is because such large‐area printing methods introduce distinct film‐drying kinetics,^[^ ^15^ ^]^ which differ from the spin‐coating method with rapid rotation. Additionally, the utilization of high‐boiling point solvents is necessary in scalable printings to ensure uniform film coverage, but this introduces complexities in the morphology evolution process and demands careful consideration.^[^ ^16^ ^]^ 3) Toxic halogenated solvents (e.g., chloroform, chlorobenzene, and *o*‐dichlorobenzene) still dominantly persist in the fabrication of state‐of‐the‐art OSCs. Despite the acknowledged need for green solvents to mitigate eco‐toxicological impacts, their adoption faces challenges in achieving optimal film morphologies.^[^ ^17^ ^]^ This challenge generally results in inferior device performance compared to their halogenated counterparts. For these reasons, the PCEs of large‐area modules fabricated with green‐solution processing significantly lag those of small‐scale hero devices. Therefore, it is of utmost importance to bridge this efficiency gap for the widespread success of large‐area modules in practical applications.

In this context, side‐chain engineering can serve a simple yet effective solution. Side‐chain modification has already been widely employed for modulating the solubility, crystallinity, and nanoscale morphology of the photoactive layer.^[^ ^18^ ^]^ Since the initial report on the high‐performance Y6 acceptor,^[^ ^6^ ^]^ various research groups have engaged in side‐chain modifications of Y6 derivatives, either in the inner pyrrole rings,^[^ ^19^, ^20^, ^21^, ^22^, ^23^, ^24^, ^25^, ^26^ ^]^ or in the *β*‐position of the outermost thiophene (or selenophene) rings of the central core.^[^ ^27^, ^28^, ^29^, ^30^, ^31^, ^32^ ^]^ For example, Lee et al. fine‐tuned the inner alkyl side chains with seven different lengths ranging from 2‐ethylhexyl (EH) to 2‐octyldodecyl (OD), and investigated their impacts on the morphological features and OSC performance.^[^ ^23^ ^]^ The subtle alteration in the inner side chains significantly improved solubility in *o*‐xylene, leading to a substantial enhancement in PCE up to 17.4% (with 2‐heptylundecyl). Meanwhile, concerning outer side chains, Sun et al. introduced branched‐type alkyl chains (2‐butyloctyl (BO), 2‐hexyldecyl (HD), and OD) to Y6 for the first time, and discovered that L8‐BO NFA exhibited optimal structural ordering and multi‐length‐scale morphology.^[^ ^27^ ^]^ Consequently, PM6:L8‐BO‐based OSCs, fabricated using chloroform, achieved a remarkable PCE of 18.32%. Despite the significant strides made by various researchers, to the best of our knowledge, their efforts have yet to culminate in a systematic study exploring the influence of both inner and outer side‐chain modification on large‐area module fabrication using a non‐halogenated solvent.

In this study, we designed and synthesized a series of Y6 analogs, including inner side chain‐modified N‐series (N‐BO, N‐HD, N‐OD) and outer side chain‐modified T‐series (T‐BO, T‐HD, T‐OD). Our aim was to systematically investigate their impact on the performance of both small‐area OSCs and large‐area modules. While a longer solubilizing group enhanced the processability of NFAs in non‐halogenated solvents, we found that the morphological and electrical properties were optimized when using the HD side chain in both the N‐ and T‐ series. As a result, by employing *o*‐xylene as the processing solvent, PM6:N‐HD‐based OSCs showed the highest PCE of 18.3% in the series, along with an outstanding fill factor (FF) of 78.7%. Most notably, we successfully fabricated large‐area (55 cm^2^) sub‐modules based on PM6:N‐HD and achieved a PCE of 12.2% in *o*‐xylene. To the best of our knowledge, this PCE ranks the highest value among all reported modules based on binary blends with an active area of over 50 cm^2^. This demonstration of a high‐efficiency large‐area module establishes N‐HD as a promising NFA for printed photovoltaic devices. To the best of our knowledge, this is the first study focusing on a comparative analysis of the effects of both inner and outer alkyl chain engineering on large‐area module fabrication using a non‐halogenated solvent.

## 2 Results and Discussion

### 2.1 NFA Design and Material Properties

**Figure 1:**
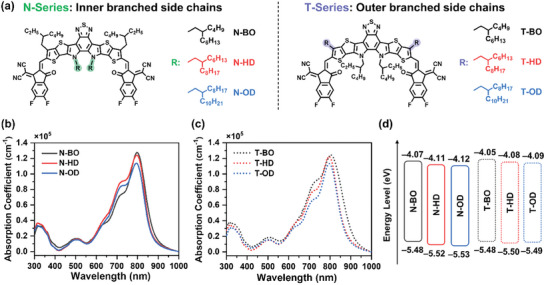
a) Molecular structures of N‐series and T‐series NFAs. Absorption coefficient spectra of b) N‐series NFAs and c) T‐series NFAs in thin films processed with *o*‐xylene. d) Energy levels of the NFAs.

The chemical structures of the NFAs are shown in **Figure** **1**a. For the N‐series, the EH side chain was selected and attached to the *β*‐position of the outermost thiophene rings of the central fused core. Then, different lengths of branched alkyl chains (i.e., BO, HD, and OD) were introduced into the pyrrole unit to obtain N‐BO, N‐HD, and N‐OD NFAs. Conversely, for the T‐series, EH was attached to the pyrrole motif, while BO, HD, and OD were attached to the *β*‐position of the outermost thiophene rings of the core to produce T‐BO, T‐HD, and T‐OD NFAs. The synthetic routes toward the NFAs are presented in Figure S1 (Supporting Information) and their successful synthesis was confirmed by ^13^C and ^1^H nuclear magnetic resonance (^13^C NMR and ^1^H NMR) and matrix‐assisted laser desorption/ionization time‐of‐flight (MALDI‐TOF) measurements (Figures S2–S7, Supporting Information).

Figure S8 (Supporting Information) shows the decomposition temperatures (*T*_d_) of the NFAs at 5% weight loss under a nitrogen atmosphere. These results indicate that all the compounds possess good thermal stability with *T*_d_ > 320 °C, which is sufficient for practical applications in OSCs. A differential scanning calorimetry (DSC) measurement was conducted to investigate the crystalline properties of the NFAs (Figure S9, Supporting Information). For both the N‐ and T‐ series, the melting temperature (*T*_m_) obtained from the exothermic peaks in the DSC curves decreased as the side‐chain length increased. The *T*_m_s of N‐BO, N‐HD, and N‐OD were 331, 287, and 239 °C, while those of T‐BO, T‐HD, and T‐OD were 317, 302, and 276 °C, respectively. The reduced crystallinity with increased alkyl‐chain length can be attributed to weakened intermolecular interactions resulting from the steric hindrance effect of longer alkyl chains.^[^ ^33^ ^]^ This can be beneficial for suppressing the excessive aggregation of NFAs during non‐halogenated solvent processing such as *o*‐xylene. Indeed, the solubility of the NFAs in *o*‐xylene greatly improved with longer side chains, increasing from 15 (N‐BO) to 134 (N‐OD) mg mL^‒1^ in the N‐series and from 18 (T‐BO) to 132 (T‐OD) mg mL^‒1^ in the T‐series (Table S1, Supporting Information).

UV–vis absorption spectroscopy was performed to investigate the optical properties of the NFAs. In the film state, the maximum absorption wavelengths (λ_max_^film^) of the BO‐based NFAs, which have the shortest side chains, were redshifted compared to those of the HD‐ and OD‐based NFAs (Figure 1b–c and **Table** **1**). Also, the maximum absorption coefficients slightly increased as the side‐chain length decreased. These characteristics can be associated with the closer intermolecular packing of NFAs with shorter side chains.^[^ ^12^ ^]^ Although N‐BO and T‐BO demonstrated favorable light harvesting ability, this did not lead to superior device performance because it is assumed that the impact of these optical properties is minimal compared to the effects of their poor blend morphologies (vide infra). In dilute *o*‐xylene solution, all the NFAs exhibited the same maximum absorption wavelength (λ_max_^soln^ = 713 nm) (Figure S10, Supporting Information; Table 1), which can be attributed to their identical conjugated backbone structure.^[^ ^34^ ^]^ However, the absorption coefficient increased in the order of OD, HD, and BO, similar to the trend observed in the film state. Cyclic voltammetry (CV) was performed to estimate the energy levels of the highest occupied molecular orbitals (HOMOs) and lowest unoccupied molecular orbitals (LUMOs) of the NFAs (Figure 1d; Figure S11, Supporting Information; Table 1). As a result, the LUMO energy levels (*E*_LUMO_) were slightly down‐shifted as the side‐chain length increased in both in the N‐series (from −4.07 to −4.12 eV) and T‐series (from −4.05 to −4.09 eV).

**Table 1:** Optical and electrochemical properties of the NFAs.

NFA	λ_max_^soln^ [nm]^a)^	λ_max_^film^ [nm]^b)^	λ_onset_ [nm]	*E*_g_^opt^ [eV]^c)^	*E*_HOMO_ [eV]^d)^	*E*_LUMO_ [eV]^d)^
N‐BO	713	798	891	1.39	−5.48	−4.07
N‐HD	713	797	879	1.41	−5.52	−4.11
N‐OD	713	794	874	1.42	−5.53	−4.12
T‐BO	713	805	918	1.35	−5.48	−4.05
T‐HD	713	796	894	1.39	−5.50	−4.08
T‐OD	713	795	887	1.40	−5.49	−4.09

^a)^ In solution state (*o*‐xylene);

^b)^ In neat films processed with *o*‐xylene;

^c)^ Optical bandgaps estimated from the absorption onset in the solid‐state: *E*_g_^opt^ = 1240/*λ*_onset_;

^d)^ Measured by cyclic voltammetry (CV) method; calculated from the equation: *E*_HOMO_ = − (*E*_ox,onset_ − *E*_1/2,ferrocene_ + 4.8) eV and *E*_LUMO_ = − (*E*_red,onset_ − *E_1_*_/2,ferrocene_ + 4.8) eV.

### 2.2 Photovoltaic Performance

**Figure 2:**
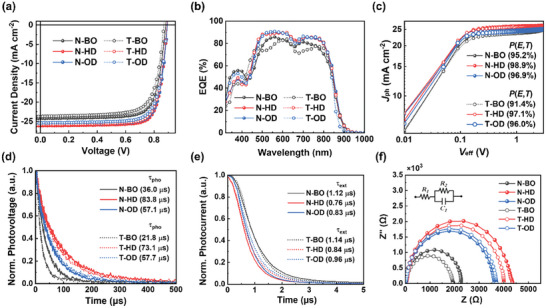
a) *J*–*V* characteristics, b) EQE spectra, c) *J*_ph_–*V*_eff_ curves, d) TPV, e) TPC graphs, and f) Nyquist plots and equivalent circuit model of PM6:NFA OSCs.

To analyze the influence of the inner/outer side chain length on device performance, OSCs were fabricated with an inverted architecture. PM6 polymer (number‐average molecular weight (*M*_n_) = 100 kg mol^−1^, polydispersity index = 3.55) was used as the donor and all the blend films were processed using *o*‐xylene without any additive. Detailed device fabrication procedures are described in the Supporting Information. The current density–voltage (*J*–*V*) characteristics of the OSC devices are illustrated in **Figure** **2**a, and the corresponding photovoltaic metrics are listed in **Table** **2**. For the N‐series, the PM6:N‐BO OSCs with the shortest BO side chains exhibited a PCE of 16.2% with an open‐circuit voltage (*V*_OC_) of 0.88 V, short‐circuit current density (*J*_SC_) of 24.2 mA cm^−2^, and FF of 75.9%. Upon introducing slightly longer HD side chains into the pyrrole motif, the PM6:N‐HD device delivered enhanced performance (PCE = 18.3%, *V*_OC_ = 0.89 V, *J*_SC_ = 26.1 mA cm^−2^, and FF = 78.7%), attributed to the improved *J*_SC_ and FF values. However, a further increase in the side‐chain length (PM6:N‐OD) resulted in a drop in the *J*_SC_ (25.7 mA cm^−2^) and PCE (17.9%) values. Meanwhile, OSCs based on the T‐series NFAs showed the same trend; the device performance was optimized with the intermediate HD side chains. For example, the PCEs of T‐BO, T‐HD, and T‐OD‐based devices were 15.2%, 17.9%, and 17.2%, respectively. These results suggest that slight modification of the inner/outer side chains of NFAs have a major impact on the blend‐film morphology processed by a non‐halogenated solvent, which will be discussed in the subsequent section.

**Table 2:** Summary of device parameters of the optimized OSCs based on PM6:NFA.

NFA	*V*_OC_ [V]	*J*_SC_ [mA cm^−2^]	*J*_SC, calc_ ^a^ ^)^ [mA cm^−2^]	FF [%]	PCE_max_ (PCE_avg_)^b^ ^)^ [%]	*µ*_e_ [cm^2^ V^−1^ s^−1^]	*µ*_h_ [cm^2^ V^−1^ s^−1^]	*µ*_h_ */µ*_e_
N‐BO	0.88	24.2	23.2	75.9	16.2 (15.5 ± 0.7)	4.15 × 10^−4^	3.09 × 10^−4^	1.34
N‐HD	0.89	26.1	25.0	78.7	18.3 (17.9 ± 0.4)	6.25 × 10^−4^	5.60 × 10^−4^	1.10
N‐OD	0.89	25.7	24.7	78.5	17.9 (17.4 ± 0.5)	4.41 × 10^−4^	3.60 × 10^−4^	1.22
T‐BO	0.87	23.7	22.8	73.9	15.2 (14.2 ± 0.9)	3.89 × 10^−4^	2.80 × 10^−4^	1.38
T‐HD	0.89	25.8	24.8	77.9	17.9 (17.3 ± 0.6)	5.93 × 10^−4^	5.01 × 10^−4^	1.18
T‐OD	0.89	25.4	24.4	76.3	17.2 (16.7 ± 0.6)	4.31 × 10^−4^	3.47 × 10^−4^	1.24

^a)^ *J*_SC_ values calculated from EQE spectra;

^b)^ PCE values in parentheses are averages obtained from over 20 devices.

As shown in the EQE spectra in Figure 2b, all OSCs exhibited broad photon responses from 300 to 925 nm, consistent with the UV–vis absorption spectra of the blend films (Figure S12, Supporting Information). The calculated *J*_SC_ (*J*_SC, calc_) values of the devices obtained from the EQE spectra were in good agreement with the *J*_SC_ values measured from the *J*–*V* curves (Table 2). To evaluate the exciton dissociation probabilities (*P*(*E*,*T*)) in these OSCs, the dependence of the photocurrent density (*J*_ph_ = *J*_L_ – *J*_D_) on the effective voltage (*V*_eff_ = *V*_0_ − *V*_a_) was measured (Figure 2c, here, *J*_L_ and *J*_D_ are the current densities under illuminated and dark conditions, respectively, while *V*_0_ is the voltage at *J*_ph_ = 0 and *V*_a_ is the applied voltage).^[^ ^35^ ^]^ In general, *J*_ph_ is linearly proportional to *V*_eff_ at a low *V*_eff_ and reaches saturation (*J*_sat_) at a high *V*_eff_, where the high internal electric field minimizes charge recombination. The *P*(*E*,*T*) value is estimated from the *J*_ph_ under short‐circuit conditions divided by *J*_sat_. Consistent with the *J*_SC_ trend, the PM6:N‐HD (98.9%) and PM6:T‐HD (97.1%) devices showed the highest *P*(*E*,*T*) values among each series, thus showing the most efficient exciton dissociation properties when employing HD side chains.

To further understand how the *J*_SC_ and FF trends correlate with the side‐chain structure, we investigated the charge recombination/extraction behaviors based on transient photovoltage (TPV) and transient photocurrent (TPC) measurements.^[^ ^36^ ^]^ The photocarrier lifetimes (*τ*_pho_) of PM6:N‐HD (83.8 µs) and PM6:T‐HD (73.1 µs), obtained from the TPV decay curves at open‐circuit conditions (Figure 2d), were significantly longer than those of the other systems. The prolonged *τ*_pho_ indicates that utilizing HD side chains both in the N‐series and T‐series can effectively suppress charge recombination in the photoactive layer.^[^ ^37^ ^]^ In addition, the charge extraction times (*τ*_ext_) of PM6:N‐HD (0.76 µs) and PM6:T‐HD (0.84 µs), obtained from the TPC analysis under the short‐circuit condition (Figure 2e), were shorter than their counterparts, implying an efficient charge extraction process.^[^ ^38^ ^]^ To conduct a more detailed analysis on the charge recombination properties, electrochemical impedance spectroscopy (EIS) was employed.^[^ ^39^, ^40^ ^]^ The Nyquist plot and equivalent resistor‐capacitor parallel circuit are presented in Figure 2f, in which *R*_1_ and *R*_2_ represent series resistance and recombination resistance, respectively. *R*_1_ and *R*_2_ can be acquired from the high‐ and low‐frequency regions of the Nyquist plots, respectively. The fitted data shows that the series resistances of all devices are nearly identical; however, the PM6:N‐HD and PM6:T‐HD systems exhibited dramatically higher recombination resistances than the other devices. Overall, these results collectively suggest that OSCs with optimized HD side chains can effectively suppress charge recombination, leading to higher *J*_SC_ and FF values.

The *V*_OC_ and *J*_SC_ profiles were measured according to light intensity (*P*_light_) to further support the recombination properties (Figure S13, Supporting Information).^[^ ^41^ ^]^ The slope (*S*) of *V*_OC_ versus *P*_light_ in Figure S13a (Supporting Information) indicates whether the dominant recombination mechanism is bimolecular (≈ 1 *kT q* ^−1^) or trap‐assisted recombination (≈ 2 *kT q* ^−1^) (where *k* is the Boltzmann constant, *T* is the Kelvin temperature, and *q* is the elementary charge). The slopes of PM6:N‐HD (*S* = 1.06 *kT q* ^−1^) and PM6:T‐HD (*S* = 1.08 *kT q* ^−1^) were determined to be smaller than the other devices in each N‐ and T‐ series. This result indicates that trap‐assisted recombination is effectively suppressed in the device, which contributes to the enhanced FF and PCE. In addition, the exponential factor (α), defined as *J*_SC_ = (*P*_light_)^α^, approaches unity when bimolecular recombination is more suppressed in the OSCs. As shown in Figure S13b (Supporting Information), the higher α values of the PM6:N‐HD (α = 0.993) and PM6:T‐HD (α = 0.989) devices than those of their counterparts indicate that the bimolecular recombination is reduced in these devices. The suppressed recombination characteristics of PM6:N‐HD and PM6:T‐HD OSCs confirmed in these experiments can be associated with the higher *J*_SC_ and FF values of the PM6:N‐HD and PM6:T‐HD OSCs compared to the other devices.

To study the charge transport properties of the blend films, electron and hole mobilities (*µ*_e_ and *µ*_h_, respectively) were measured using the space‐charge‐limited current (SCLC) method (Figure S14, Supporting Information; Table 2).^[^ ^42^ ^]^ The PM6:N‐BO device exhibited the lowest mobilities (*µ*_e_ = 4.15 × 10^−4^ cm^2^ V^−1^ s^−1^ and *µ*_h_ = 3.09 × 10^−4^ cm^2^ V^−1^ s^−1^) among the N‐series, as well as a relatively unbalanced charge transport (*µ*_e_/*µ*_h_ = 1.34). This result can be attributed to the low solubility and high aggregation tendency of N‐BO, resulting in severe charge recombination and reduction in *J*_SC_ and FF. By contrast, the PM6:N‐HD device achieved the highest mobilities (*µ*_e_ = 6.25 × 10^−4^ cm^2^ V^−1^ s^−1^ and *µ*_h_ = 5.60 × 10^−4^ cm^2^ V^−1^ s^−1^), as well as the most balanced characteristics (*µ*_e_/*µ*_h_ = 1.10). However, further increasing the chain length at the inner position worsened the charge transport behaviors (i.e., *µ*_e_ = 4.41 × 10^−4^ cm^2^ V^−1^ s^−1^ and *µ*_h_ = 3.60 × 10^−4^ cm^2^ V^−1^ s^−1^ for PM6:N‐OD). The T‐series showed the same trend with that of the N‐series, where optimized charge transport properties were observed with the intermediate HD outer side chains. This mobility trend can be understood by examining the morphological aspects discussed in the next section. These results correlate with the lower charge recombination and more efficient charge extraction of the PM6:N‐HD and PM6:T‐HD devices, as discussed above.

### 2.3 Morphology Investigation

**Figure 3:**
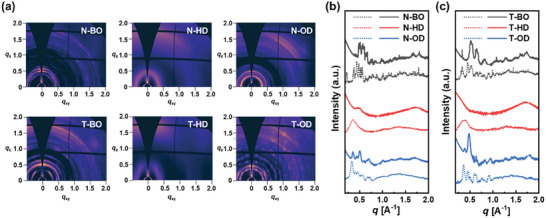
a) 2D‐GIWAXS pattern images of N and T‐series neat films; Line‐cut profiles of b) N‐ and c) T‐series neat films along the IP (dotted lines) and OOP (solid lines) directions.

Grazing‐incidence wide‐angle X‐ray scattering (GIWAXS) measurements were conducted to investigate the intermolecular packing in thin film.^[^ ^43^ ^]^ In the neat NFA films (**Figure** **3**), only the N‐HD and T‐HD films exhibited a clear lamellar stacking (100) peak in the in‐plane (IP) direction and π–π stacking (010) peak in the out‐of‐plane (OOP) direction. These scattering patterns indicate that the N‐HD and T‐HD crystallites have face‐on orientation, which is favorable for charge transport in the vertical direction.^[^ ^44^ ^]^ By stark contrast, the introduction of BO and OD side chains at both the inner and outer positions of NFA led to the formation of multiple diffraction patterns with orientational disorder, as confirmed by several Debye rings.^[^ ^45^, ^46^ ^]^ These results show that a subtle change in the inner/outer NFA side chains can greatly impact the crystalline nature in thin film.

Upon blending with the PM6 donor, all the blend films showed similar scattering patterns with dominantly face‐on orientation (Figure S15 and Table S2, Supporting Information). We note that the multiple diffraction patterns observed in the neat BO‐ and OD‐based NFAs disappeared, which may be attributed to the earlier crystallization of PM6, guiding the crystallization process of the NFAs (vide infra).^[^ ^10^ ^]^ The interlamellar distances (*d*_100_) of the blend films were in good agreement with the sequential extension of the side chains (BO → HD → OD) for both the N‐series (18.6 → 19.6 → 19.9 Å) and T‐series (19.5 → 20.7 → 21.2 Å). In addition, the π–π stacking distance (*d*_π–π_) of the NFAs increased with the lengthened side chains for both the N‐series (3.647 → 3.718 → 3.744 Å) and T‐series (3.674 → 3.724 → 3.771 Å), which is likely due to the steric hindrance effect of bulkier side chains.^[^ ^47^ ^]^ To note, the smaller *d*_π–π_ values of the N‐series than those of the T‐series are consistent with the higher SCLC mobility values of the N‐series (Table 2). However, the mobility trend according to side‐chain length does not align well. For example, although the BO‐based NFAs demonstrated the smallest *d*_π–π_ values in each series, their mobilities were the lowest. This is because the changes in blend morphology depending on the side‐chain length are significant (vide infra).

**Figure 4:**
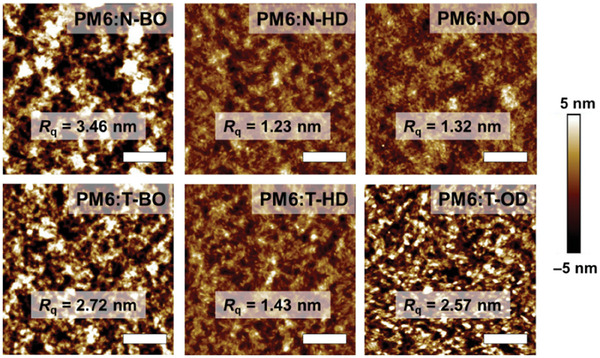
AFM height images of PM6:NFA blend films. The scale bars indicate 500 nm.

Atomic force microscopy (AFM) was performed to investigate the influence of inner and outer branched alkyl‐chain length on the photoactive layer surface morphology (**Figure** **4**). The root‐mean‐square roughness (*R*_q_) values of all photoactive blend films varied depending on the alkyl‐chain length. Among the N‐series, the PM6:N‐BO blend film showed the largest roughness (*R*_q_ = 3.46 nm), which can be attributed to the aggregation tendency of N‐BO under *o*‐xylene processing.^[^ ^48^ ^]^ As the length of the alkyl chain increased, the PM6:N‐HD blend film exhibited a smoother surface (*R*_q_ = 1.23 nm). In general, smooth surface is advantageous for charge transfer and extraction from the photoactive layer to the electrode,^[^ ^49^, ^50^, ^51^, ^52^, ^53^ ^]^ which is also consistent with the *τ*_ext_ trend in the TPC analysis. However, as observed in the PM6:N‐OD blend film, a further increase in the alkyl‐chain length rather resulted in a slightly higher roughness (*R*_q_ = 1.32 nm), which may have been one of the factors that reduced *J*_SC_. The T‐series exhibited a similar trend to the N‐series; PM6:T‐BO, PM6:T‐HD, and PM6:T‐OD showed *R*_q_ values of 2.72, 1.43, and 2.57 nm, respectively. Consequently, selecting an optimized alkyl‐chain length for both the inner and outer positions can lead to the formation of a smooth surface, ultimately enhancing the charge extraction.

**Figure 5:**
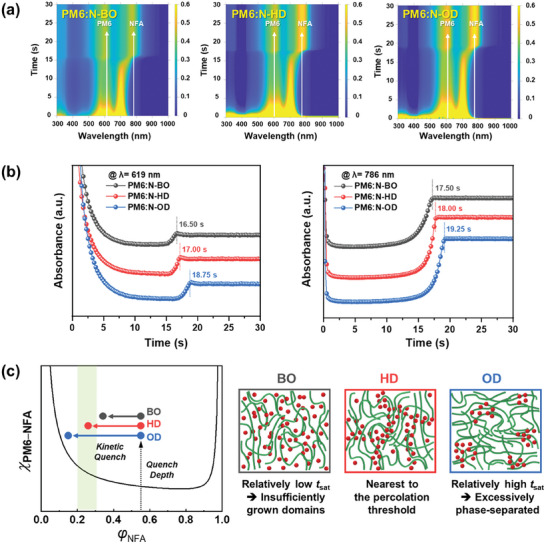
a) 2D contour maps of PM6:N‐BO, PM6:N‐HD, and PM6:N‐OD blend films obtained by in situ UV–vis spectroscopy and their b) line‐cut profiles at λ = 619 and 786 nm. c) Phase diagram and schematic morphologies conceptually illustrating kinetic quench differences of the BO‐, HD‐, and OD‐based NFA blends with PM6. χ_PM6–NFA_ indicates Flory‐Huggins interaction parameter and *φ*_NFA_ indicates volume composition of NFA. The region with green vertical shading in the phase diagram marks the vicinity of percolation threshold for charge transport.

To more deeply understand the morphological features of the PM6:NFA blend films, the morphology evolution process during film formation was explored by in situ UV–vis spectroscopy.^[^ ^15^ ^]^ Absorbance of the PM6:NFA blend films was monitored as a function of spin coating time, and the resultant 2D contour maps and line‐cut profiles are shown in **Figure** **5**a,b (for the PM6:N‐series) and Figure S16 (Supporting Information) (for the PM6:T‐series) (here, λ = 619 and 786 nm correspond to the λ_max_ of PM6 and NFAs, respectively). For all the blend films, it was observed that the PM6 donor crystallized earlier than the NFAs. This result may be correlated with the GIXS data: the reason why the multiple diffraction patterns observed in the neat BO or OD films disappeared in the blend films may be explained by the preceding crystallization of PM6, potentially influencing the crystallization of the NFAs.^[^ ^10^ ^]^ In addition, the saturation time (*t*_sat_) of absorbance increased as the side‐chain length increased. For example, the *t*_sat_ of PM6:N‐BO, PM6:N‐HD, and PM6:N‐OD showed a sequential increase of 16.50, 17.00, and 18.75 s at λ = 619 nm and 17.50, 18.00, and 19.25 s at λ = 786 nm, respectively. This result is consistent with previous reports in which a higher solubility of photoactive materials induced a slower precipitation of the liquid state, thereby leading to longer *t*_sat_.^[^ ^54^, ^55^ ^]^

Based on these data, a phase diagram and schematic illustration are suggested as in Figure 5c to grasp the correlation between the film‐formation kinetics and morphology of the PM6:NFA blend films.^[^ ^56^, ^57^ ^]^ For the estimation of quench depth, contact‐angle measurement was performed (Figure S17 and Table S3, Supporting Information), and the interfacial tension (*γ*_PM6–NFA_) was employed to determine the order between the Flory‐Huggins interaction parameters (χ_PM6–NFA_) of each blend.^[^ ^58^ ^]^ Overall, we propose that the degree of kinetic quench, which varied depending on the side‐chain length, was a decisive factor affecting the morphology as follows. For the NFAs with the shortest BO side chains, the morphology was rapidly quenched due to their low solubility, reducing the duration of the liquid–liquid phase separation.^[^ ^59^ ^]^ Thus, overly intermixed blend morphologies with insufficiently grown domains were formed, leading to severe charge recombination and inefficient charge transport. By contrast, for the NFAs with the longest OD side chains, the time for morphology evolution was prolonged due to their high solubility. This led to excessively phase‐separated morphologies, hindering charge generation. Meanwhile, the intermediate *t*_sat_ values of PM6:N‐HD and PM6:T‐HD blend films led to an ideal morphology with proper domain sizes, which is presumed to be nearest to the percolation threshold for charge transport, as confirmed in the SCLC results. This explanation can be also partially supported by transmission electron microscopy (TEM) images of the PM6:NFA blend films (Figure S18, Supporting Information), where the size of dark regions and phase contrast were most pronounced in the OD‐based samples, suggesting enhanced phase separation. Therefore, we conclude that these morphological features, depending on the side chains, have resulted in the photovoltaic performance trend discussed above.

### 2.4 Large‐Area Sub‐Module Fabrication

**Figure 6:**
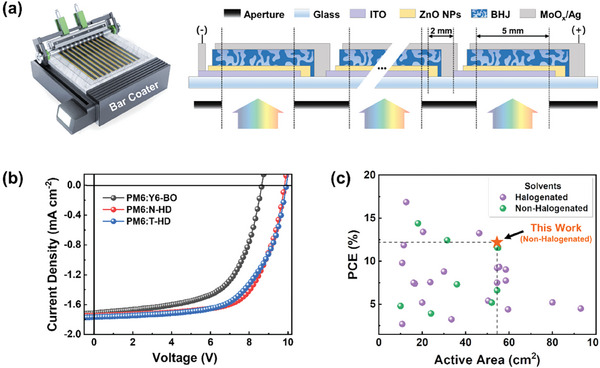
a) Schematic illustration of a bar coater and cross‐sectional diagram of a large‐area sub‐module. b) *J*–*V* characteristics of PM6:Y6‐BO, PM6:N‐HD, and PM6:T‐HD sub‐modules. c) PCE versus active area graph for reported sub‐modules in literature processed by using halogenated or non‐halogenated solvents.

To confirm the applicability of our NFAs into large‐area device fabrication for future commercialization, we fabricated PM6:N‐HD and PM6:T‐HD‐based sub‐modules (substrate size: 12 × 12 cm^2^, photoactive area: 55 cm^2^) consisting of 11 cells connected in series arrangement (**Figure** **6**a). The sub‐modules were fabricated with bar coating in the air at room temperature, and the detailed procedures for their fabrication are presented in the Supporting Information. As a reference system, Y6‐BO, which is one of the representative high‐performance NFAs used for non‐halogenated solution processing,^[^ ^21^ ^]^ was employed. The *J*–*V* curves of each system are shown in Figure 6b and their photovoltaic parameters are presented in **Table** **3**. Among the three systems, the PM6:N‐HD sub‐module provided the best PCE of 12.2% (*V*_OC_ = 9.88 V, *J*_SC_ = 1.85 mA cm^−2^, and FF = 67.0%). Notably, this PCE is one of the highest values reported in the literature to date for large‐area sub‐modules processed in non‐halogenated solvents at ambient conditions (Figure 6c; Table S4, Supporting Information). It is also noteworthy that an encapsulated PM6:N‐HD‐based sub‐module exhibited good thermal stability in air by maintaining more than 93% of the initial PCE after 300 h at 80 °C (Figure S19, Supporting Information). Meanwhile, the PM6:T‐HD‐based sub‐module yielded a slightly lower PCE of 11.4% (*V*_OC_ = 9.77 V, *J*_SC_ = 1.75 mA cm^–2^, and FF = 66.5%) and the PM6:Y6‐BO reference system exhibited a much lower PCE of 9.4% (*V*_OC_ = 9.17 V, *J*_SC_ = 1.60 mA cm^−2^, and FF = 63.6%). Consequently, our results, based on the alkyl‐chain‐optimized NFAs, offer a foundation for the development of high‐performance sub‐modules fabricated using non‐halogenated solution processing at room temperature. Furthermore, this work presents a roadmap for the transition from small‐scale devices to roll‐to‐roll (R2R)‐processed large‐area modules.

**Table 3:** Photovoltaic performance of large‐area (photoactive area = 55 cm^2^) sub‐modules.

Photoactive Layer	*V*_OC_ [V]	*J*_SC_ [mA cm^−2^]	FF [%]	PCE_max_ (PCE_avg_)^a)^ [%]
PM6:Y6‐BO	9.17	1.60	63.6	9.4 (8.2 ± 1.3)
PM6:N‐HD	9.88	1.85	67.0	12.2 (11.6 ± 0.6)
PM6:T‐HD	9.77	1.75	66.5	11.4 (10.7 ± 0.7)

^a)^ PCEs in parentheses are average values obtained from over 5 sub‐modules.

## 3 Conclusion

Our investigation into inner‐side chain‐modulated (N‐series) and outer‐side chain‐modulated (T‐series) Y6 derivatives has provided insights into their impact on the photovoltaic performance of both small‐area OSCs and large‐area sub‐modules processed with *o*‐xylene. Variations in side‐chain length in the inner/outer positions of NFAs greatly influenced the aggregation characteristics, solubility, and film‐formation kinetics. Consequently, we found that the blend morphology and electrical properties of PM6:NFA were optimized when having intermediate HD side chains in both the N‐series (N‐HD) and T‐series (T‐HD), as confirmed by a set of analyses such as AFM, GIWAXS, and SCLC measurements. Accordingly, the PM6:N‐HD‐based OSCs achieved the highest PCE of 18.3% with an impressive FF of 78.7%. The superior performance of PM6:N‐HD was attributed to efficient exciton dissociation and suppressed charge recombination, as a result of its favorable blend morphology. On the basis of these results, large‐area sub‐modules (photoactive area: 55 cm^2^) were also fabricated based on the PM6:N‐HD system in the ambient condition with *o*‐xylene at room temperature. As a result, the sub‐modules achieved a PCE of 12.2%, which ranks among the highest values reported to date. This research demonstrates that employing inner/outer side‐chain engineering in NFAs represents an effective approach in developing highly efficient large‐area sub‐modules based on non‐halogenated solvent processing.

## Conflict of Interest

The authors declare no conflict of interest.

## Supporting information

Supporting Information

## Data Availability

The data that support the findings of this study are available on request from the corresponding author. The data are not publicly available due to privacy or ethical restrictions.
