# Supplementary material for: Inner/Outer Side Chain Engineering of Non‐Fullerene Acceptors for Efficient Large‐Area Organic Solar Modules Based on Non‐Halogenated Solution Processing in Air
Source: Adv Sci (Weinh). 2024 Jul 16;11(35):2405716. doi: 10.1002/advs.202405716 (PMC11425251; doi:10.1002/advs.202405716)
Supplement: Supplementary file 1 — Supporting Information [file ADVS-11-2405716-s001.docx]

Supporting Information

Inner/Outer Side Chain Engineering of Non-Fullerene Acceptors for Efficient Large-Area Organic Solar Modules Based on Non-Halogenated Solution Processing in Air

Sabeen Zahra†, Seungjin Lee†, Muhammad Jahankhan†, Muhammad Haris, Du Hyeon Ryu, Bumjoon J. Kim, Chang Eun Song*, Hang Ken Lee, Sang Kyu Lee and Won Suk Shin*

S. Zahra, Dr. S. Lee, M. Jahankhan, M. Haris, D. H. Ryu, Dr. C. E. Song, Dr. H. K. Lee, Dr. S. K. Lee, Dr. W. S. Shin

Advanced Energy Materials Research Center, Korea Research Institute of Chemical Technology (KRICT), Daejeon 34114, Republic of Korea

E-mail: songce@krict.re.kr, shinws@krict.re.kr

S. Zahra, M. Jahankhan, M. Haris, Prof. C. E. Song, Prof. H. K. Lee, Prof. S. K. Lee, Prof. W. S. Shin

Advanced Materials and Chemical Engineering, University of Science and Technology (UST), Daejeon 34113, Republic of Korea

Prof. B. J. Kim

Department of Chemical and Biomolecular Engineering, Korea Research Institute of Science and Technology (KAIST), Daejeon 34141, Republic of Korea

†These authors contributed equally to this work.

*Corresponding authors: songce@krict.re.kr (C. E. Song) and shinws@krict.re.kr (W. S. Shin)

**Experimental Details**

**Materials and measurement**

All commercially available reagents and solvents were directly used without further purification unless otherwise specified. PM6 polymer donor was purchased from Derthon Optoelectronics Materials Science Technology Co LTD. The number-average molecular weight (*M*_n_) and polydispersity index of PM6 were 100 kg mol^−1^ and 3.55, respectively, which were measured using gel permeation chromatography (GPC) with chloroform eluent relative to polystyrene standards. ^1^H NMR (400 MHz) and ^13^C NMR (100MHz) spectra were recorded on Bruker AVANCE III HD 400 MHz NMR spectrometer in CDCl_3_. Matrix assisted laser desorption/ionization time-of-flight (MALDI-TOF) mass spectra were tested on a Bruker Autoflex instrument. Thermogravimetric analysis (TGA) was performed using Thermo plus EVOII TG8120, samples were run under N_2_ and heated from room temperature to 600 °C at a rate of 10 °C min^−1^. UV-vis absorption measurements were carried out with a double-beam Shimadzu UV-2550 spectrophotometer over 300–1000 nm. The UV–Vis spectra in solution were obtained in dilute *o*-xylene solution (1.0 × 10^−5^ M), while the UV–Vis spectra in thin film were obtained by spin-coating *o*-xylene solution (15 mg mL^−1^) onto quartz substrates. Cyclic voltammetry (CV) was measured by using an IviumStat instrument and conducted at a scan rate of 50 mV s^−1^ at 25 °C under argon with 0.1 M tetrabutylammonium hexafluorophosphate in acetonitrile as the electrolyte. Small molecules were deposited onto the working electrode from chloroform solution. All CV measurements were carried out at room temperature with a conventional three-electrode configuration employing a glassy carbon electrode as the working electrode, a saturated calomel electrode as the reference electrode, and a Pt wire as the counter electrode. The highest occupied molecular orbital (HOMO) and lowest unoccupied molecular orbital (LUMO) energy levels were calculated from the onset oxidation potential and the onset reduction potential, using the empirical relationship *E*_HOMO_ = − (*E*_onset,ox_ − *E*_1/2,ferrocene_ + 4.8) eV and *E*_LUMO_ = −(*E*_onset,red_ − *E*_1/2,ferrocene_ + 4.8) eV.

**Solubility measurement**

1 mL of *o*-xylene was added to 150 mg of each NFA. The solution was stirred at room temperature for 2 h and filtered through a 0.2 μm PTFE membrane. 0.5 mL of the filtrate was taken and methanol was slowly added until all the solids precipitated. The precipitate was then filtered and dried under vacuum. The solubility of the NFAs was calculated by the following equation.

$$\text{S}\text{olubility (mg}\text{ }\text{mL}\text{‒1}\text{) =} \frac{\text{W}\text{eight of the precipitate (mg)}}{\text{0.5 mL}}$$

**Synthetic procedure details**

The synthetic routes toward non-fullerene acceptor N and T-series with branched alkyl chains are shown in **Figure** **S1**. Compound 1-N and 1-T was synthesized by the procedure in literature,^1^ followed by the reaction with R-Br under alkaline conditions. The Vilsmeier-Haack reaction of compound 2-N and 2-T with POCl_3_ and DMF generated compound 3-N and 3-T, which was further converted to the desired compound N-R and T-R via the Knoevenagel condensation reaction of compound 3-N and 3-T with 2-(5,6-difluoro-3-oxo-2,3-dihydro-1H-inden-1-ylidene)malononitrile.

*Synthesis of compound 2-N*

A mixture of 1-N (0.6 g, 0.904 mmol), R-Br (4.5 mmol), potassium carbonate (0.99 g, 7.17 mmol), and potassium iodide (30 mg), in DMF was stirred overnight at 90 °C. After cooling to room temperature, reaction mixture was poured into 40 mL water and extracted with dichloromethane. Combined organic layer was dried over anhydrous MgSO_4_, filtrated, and concentrated under reduced pressure. The residue was purified with column chromatography on silica gel using a mixture solvent of hexane and dichloromethane 7:1 as an eluent to give compound 2-N.

*Compound BO:*

The starting material compound R-Br should be compound 2-butyl octyl bromide (1.12 g, 4.5 mmol). The final product compound was obtained as an orange-yellow solid (0.5 g, 55%). ^1^H NMR (400 MHz, CDCl_3_, δ): 6.99 (s, 2H), 4.61-4.59 (d, 4H), 2.76-2.74 (d, 4H), 2.06-2.00 (m, 4H), 1.35-1.26 (m, 36H), 0.91-0.87 (m, 24H), 0.66-0.58 (m, 12H).

*Compound HD:*

The starting material compound R-Br should be compound 2-hexyl decyl bromide (1.38 g, 4.5 mmol). The final product compound was obtained as a fluorescent yellow solid (0.5 g, 51 %). ^1^H NMR (400 MHz, CDCl_3_, δ): 6.98 (s, 2H), 4.61-4.59 (d, 4H), 2.76-2.74 (d, 4H), 2.06-1.99 (m, 4H), 1.35-1.26 (m, 52H), 0.87-0.85 (m, 24H), 0.64-0.60 (m, 12H).

*Compound OD:*

The starting material compound R-Br should be compound 2-octyl dodecyl bromide (1.63 g, 4.5 mmol). The final product compound was obtained as an orange solid (0.5 g, 45 %). ^1^H NMR (400 MHz, CDCl_3_, δ): 6.98 (s, 2H), 4.61-4.59 (d, 4H), 2.75-2.74 (d, 4H), 2.06-1.99 (m, 4H), 1.35-1.26 (m, 68H), 0.88-0.85 (m, 24H), 0.66-0.57 (m, 12H).

*Synthesis of compound 3-N*

To a solution of 2-N (0.4 mmol) in 1,2-dichloroethane (25 mL), DMF (0.4 mL) was added under the protection of argon. The reaction mixture was then cooled to 0 °C, followed by slow addition of POCl_3_ (1 mL). After being kept at 0 ^o^C for 1 h, the reaction mixture was heated to 70 ^o^C and stirred for 6 h. After cooled to room temperature, the reaction mixture was quenched with NaHCO_3_ solution and extracted with dichloromethane. The combined extracts were washed with brine, dried over anhydrous MgSO_4_, and then filtered. The solvent was removed by rotary evaporation to yield the crude product. The obtained crude product was further purified by column chromatography on silica gel with a mixture solvent of hexane and dichloromethane 1:1 as an eluent to afford compound 3-N.

*Compound BO:*

The starting material compound 2-N should be compound BO (0.40 g, 0.4 mmol). The final product compound was obtained as a bright yellow solid (0.35 g, 84 %). ^1^H NMR (400 MHz, CDCl_3_, δ): 10.13 (s, 2H), 4.65-4.63 (d, 4H), 3.12-3.10 (d, 4H), 2.08-2.00 (m, 4H), 1.38-1.26 (m, 36H), 0.88-0.86 (m, 24H), 0.70-0.65 (m, 6H), 0.61-0.56 (m, 6H).

*Compound HD:*

The starting material compound 2-N should be compound HD (0.44 g, 0.4 mmol). The final product compound was obtained as a bright yellow solid (0.39 g, 84 %). ^1^H NMR (400 MHz, CDCl_3_, δ): 10.12 (s, 2H), 4.65-4.63 (d, 4H), 3.12-3.10 (d, 4H), 2.08-2.00 (m, 4H), 1.38-1.26 (m, 52H), 0.96-0.93 (m, 12H), 0.86-0.84 (m, 12H),0.70 (t, 6H), 0.68-0.66 (t, 6H).

*Compound OD:*

The starting material compound 2-N should be compound OD (0.49 g, 0.4 mmol). The final product compound was obtained as a bright yellow solid (0.43 g, 84 %). ^1^H NMR (400 MHz, CDCl_3_, δ): 10.12 (s, 2H), 4.65-4.63 (d, 4H), 3.12-3.10 (d, 4H), 2.07-2.00 (m, 4H), 1.38-1.25 (m, 68H), 0.95-0.93 (m, 12H), 0.87-0.84 (m, 12H), 0.7-0.68 (t, 6H), 0.67-0.65 (t, 6H).

*Synthesis of compound N-R*

Under nitrogen protection, compound 3-N (0.1 mmol), 2-(5,6-difluoro-3-oxo-2,3-dihydro-1H-inden-1-ylidene)malononitrile (85 mg, 0.4 mmol) and chloroform (20 mL) was added to 50 mL two-necked round bottom flask. After the reaction mixture was stirred at room temperature for 10 mins, pyridine (0.4 mL) was then added. The reaction mixture was allowed to stir at 65 ºC for 8 h. After removal of solvent of reaction mixture, methanol was added and the precipitate was collected by filtration to get crude product, which was further purified by column chromatography on silica gel with hexane: dichloromethane (1:1) as an eluent to afford compound N-R.

*Compound N-BO:*

The starting material compound 3-N should be compound BO (100 mg, 0.1 mmol). The final product compound N-BO was obtained as a dark solid (120 mg, 81 %). ^1^H NMR (400 MHz, CDCl_3_, δ): 9.15 (s, 2H), 8.58-8.54 (m, 2H), 7.71-7.68 (t, 2H), 4.78-4.77 (d, 4H), 3.19-3.17 (d, 4H), 2.15-2.02 (m, 4H), 1.53-1.01 (m, 36H), 1.01-0.94 (m, 12H), 0.94-0.86 (m, 12H), 0.83-0..68 (t, 6H), 0.67 (t, 6H). ^13^C NMR (400 MHz, CDCl_3_, δ): 186.00, 159.06, 155.61, 153.44, 153.04, 147.53, 145.38, 137.55, 135.76, 135.67, 134.57, 134.19, 134.09, 130.97, 120.11, 115.10, 114.84, 114.58, 113.51, 112.50, 112.31, 68.77, 55.73, 41.47, 39.20, 34.44, 32.71, 31.56, 30.45, 29.38, 28.80, 27.97, 27.84, 26.03, 25.49,25.34, 23.01, 22.82, 22.78, 22.44, 14.07, 13.99, 13.75, 13.72, 10.98. MS (MALDI-TOF) m/z: calcd 1478.57, found M^+^: 1478.525.

*Compound N-HD:*

The starting material compound 3-N should be compound HD (117 mg, 0.1 mmol). The final product compound N-HD was obtained as a dark solid (110.2 mg, 69 %). ^1^H NMR (400 MHz, CDCl_3_, δ): 9.16 (s, 2H), 8.60-8.56 (m, 2H), 7.72-7.68 (t, 2H), 4.77-4.75 (d, 4H), 3.21-3.19 (d, 4H), 2.13-2.02 (m, 4H), 1.44-1.02 (m, 52H), 0.98-0.95 (m, 12H), 0.93-0.87 (m, 12H), 0.82-0.78 (t, 6H), 0.68-0.64 (t, 6H). ^13^C NMR (400 MHz, CDCl_3_, δ): 185.99, 159.10, 155.77, 153.44, 153.13, 147.53, 146.40, 145.39, 137.56, 135.72, 134.52, 134.22, 134.11, 131.02, 120.11, 119.17, 115.09, 114.88, 114.59, 113.52, 112.50, 68.79, 55.72, 41.48, 39.20, 34.45, 32.72, 31.84, 31.56, 30.61, 29.74, 29.34, 29.18, 28.80, 26.03, 23.02, 22.61, 22.45, 14.08, 13.99, 10.97. MS (MALDI-TOF) m/z: calcd 1590.70, found M^+^ : 1590.617.

*Compound N-OD:*

The starting material compound 3-N should be compound OD (128 mg, 0.1 mmol). The final product compound N-OD was obtained as a dark solid (136 mg, 80 %).^1^H NMR (400 MHz, CDCl_3_, δ): 9.16 (s, 2H), 8.59-8.55 (q, 2H), 7.72-7.68 (t, 2H), 4.78-4.76 (d, 4H), 3.20-3.18 (d, 4H), 2.13-2.02 (m, 4H), 1.53-1.00 (m, 68H), 0.99-0.96 (m, 12H), 0.94-0.88 (m, 12H), 0.87-0.83 (t, 6H), 0.81-0.78 (t, 6H). 13C NMR (400 MHz, CDCl_3_, δ): 185.98, 159.07, 155.75, 153.45, 147.52, 145.40, 137.59, 136.68, 135.76, 134.64, 134.58, 134.20, 134.11, 130.99, 120.09, 115.08, 114.86, 114.58, 113.55, 112.49, 112.31, 68.82, 55.74, 41.48, 39.17, 34.45, 32.73, 31.92, 31.83, 30.59, 29.73, 29.62, 29.53, 29.41, 29.33, 29.18, 28.80, 26.04, 25.60, 23.02, 22.68, 22.61, 14.08, 10.98. MS (MALDI-TOF) m/z: calcd 1702.82, found M^+^: 1702.66.

*Synthesis of compound 2-T*

A mixture of 1-T (0.77 mmol), 2-ethylhexyl bromide (0.75 g, 3.86 mmol), potassium carbonate (0.85 g, 6.15 mmol), and potassium iodide (30 mg), in DMF was stirred overnight at 90 °C. After cooling to room temperature, reaction mixture was poured into 40 mL water and extracted with dichloromethane. Combined organic layer was dried over anhydrous magnesium sulfate (MgSO_4_) and then concentrated. The crude product was purified by silica gel column using a mixture solvent of hexane and dichloromethane 6:1 as an eluent to give compound 2-T.

*Compound BO:*

The starting material compound 1-T should be compound BO (0.6 g, 0.77 mmol). The final product compound was obtained as an orange yellow solid (0.48 g, 60%). ^1^H NMR (400 MHz, CDCl_3_, δ): 6.99 (s, 2H), 4.61-4.59 (d, 4H), 2.76-2.74 (d, 4H), 2.06-2.00 (m, 4H), 1.35-1.26 (m, 36H), 0.91-0.87 (m, 24H), 0.66-0.58 (m, 12H).

*Compound HD:*

The starting material compound 1-T should be compound HD (0.68 g, 0.77 mmol). The final product compound was obtained as a fluorescent yellow solid (0.48 g, 55 %). ^1^H NMR (400 MHz, CDCl_3_, δ): 6.98 (s, 2H), 4.61-4.59 (d, 4H), 2.76-2.74 (d, 4H), 2.06-1.99 (m, 4H), 1.35-1.26 (m, 52H), 0.87-0.85 (m, 24H), 0.64-0.60 (m, 12H).

*Compound OD:*

The starting material compound 1-T should be compound OD (0.77 g, 0.77 mmol). The final product compound was obtained as an orange solid (0.5 g, 52 %). ^1^H NMR (400 MHz, CDCl_3_, δ): 6.98 (s, 2H), 4.61-4.59 (d, 4H), 2.75-2.74 (d, 4H), 2.06-1.99 (m, 4H), 1.35-1.26 (m, 68H), 0.88-0.85 (m, 24H), 0.66-0.58 (m, 12H).

*Synthesis of compound 3-T*

To a solution of 2-T (0.4 mmol) in 1,2-dichloroethane (25 mL), DMF (0.5 mL) was added under the protection of argon. The reaction mixture was then cooled to 0 °C, followed by slow addition of POCl_3_ (1 mL). After being kept at 0 ^o^C for 1 h, the reaction mixture was heated to 70 °C and stirred for 6 h. After cooled to room temperature, the reaction mixture was quenched with NaHCO_3_ solution and extracted with dichloromethane. The combined extracts were washed with brine, dried over anhydrous MgSO_4_, and then filtered. The solvent was removed by rotary evaporation to yield the crude product. The obtained crude product was further purified by column chromatography on silica gel with a mixture solvent of hexane and dichloromethane 1:1 as an eluent to afford compound 3-T.

*Compound BO:*

The starting material compound 2-T should be compound BO (0.40 g, 0.4 mmol). The final product compound was obtained as a bright yellow solid (0.36 g, 85 %). ^1^H NMR (400 MHz, CDCl_3_, δ): 10.13 (s, 2H), 4.65-4.63 (d, 4H), 3.12-3.10 (d, 4H), 2.08-2.00 (m, 4H), 1.38-1.26 (m, 36H), 0.88-0.86 (m, 24H), 0.70-0.65 (m, 6H), 0.61-0.56 (m, 6H).

*Compound HD:*

The starting material compound 2-T should be compound HD (0.44 g, 0.4 mmol). The final product compound was obtained as a bright yellow solid (0.38 g, 81 %). ^1^H NMR (400 MHz, CDCl_3_, δ): 10.12 (s, 2H), 4.65-4.63 (d, 4H), 3.12-3.10 (d, 4H), 2.07-1.99 (m, 4H), 1.40-1.27 (m, 52H), 0.97-0.94 (m, 12H), 0.87-0.85(m, 12H),0.70 (t, 6H), 0.69-0.67 (t, 6H).

*Compound OD:*

The starting material compound 2-T should be compound OD (0.49 g, 0.4 mmol). The final product compound was obtained as a bright yellow solid (0.43 g, 84%). ^1^H NMR (400 MHz, CDCl_3_, δ): 10.12 (s, 2H), 4.65-4.63 (d, 4H), 3.12-3.10 (d, 4H), 2.07-2.00 (m, 4H), 1.39-1.26 (m, 68H), 0.94-0.92 (m, 12H), 0.88-0.85 (m, 12H), 0.70-0.68 (t, 6H), 0.67-0.65 (t, 6H).

*Synthesis of compound T-R*

Under nitrogen protection, compound 3-T (0.1 mmol), 2-(5,6-difluoro-3-oxo-2,3-dihydro-1H-inden-1-ylidene)malononitrile (85 mg, 0.4 mmol) and chloroform (25 mL) was added to 50 mL two-necked round bottom flask. After the reaction mixture was stirred at room temperature for 10 mins, pyridine (0.4 mL) was then added. The reaction mixture was allowed to stir at 65 ºC for 8 h. After removal of solvent of reaction mixture, methanol was added and the precipitate was collected by filtration to get crude product, which was further purified by column chromatography on silica gel with hexane: dichloromethane (1:1) as an eluent to afford compound T-R.

*Compound T-BO:*

The starting material compound 3-T should be compound BO (100 mg, 0.1 mmol). The final product compound T-BO was obtained as a dark solid (120 mg, 81 %). ^1^H NMR (400 MHz, CDCl_3_, δ): 9.12 (s, 2H), 8.56-8.52 (m, 2H), 7.72-7.68 (t, 2H), 4.81-4.79 (d, 4H), 3.16-3.14 (d, 4H), 2.12-2.05 (m, 4H), 1.48-1.15 (m, 36H), 1.11-0.92 (m, 12H), 0.88-0.80 (m, 12H), 0.79-0.78 (t, 6H), 0.67 (t, 6H). ^13^C NMR (400 MHz, CDCl_3_, δ): 186.04, 158.99, 153.54, 147.57, 145.32, 137.65, 136.66, 135.74, 135.63, 134.47, 134.04, 133.88, 130.69, 120.06, 115.12, 115.02, 114.80, 114.56, 113.59, 112.54, 112.36, 68.75, 55.69, 40.41, 40.05, 34.76, 33.60, 33.34, 31.88, 29.68, 29.63, 28.86, 27.65, 26.59, 23.29, 23.00, 22.82, 22.65, 14.08, 14.05, 13.73, 10.27. MS (MALDI-TOF) m/z: calcd 1478.57, found M^+^: 1478.535.

*Compound T-HD:*

The starting material compound 3-T should be compound HD (117 mg, 0.1 mmol). The final product compound T-HD was obtained as a dark solid (112 mg, 70 %). ^1^H NMR (400 MHz, CDCl_3_, δ): 9.13 (s, 2H), 8.57-8.53 (m, 2H), 7.72-7.68 (t, 2H), 4.80-4.78 (d, 4H), 3.17-3.15 (d, 4H), 2.12-2.08 (m, 4H), 1.40-1.21 (m, 52H), 1.03-1.00 (m, 12H), 0.84-0.81 (m, 12H), 0.79-0.75 (t, 6H), 0.68-0.65 (t, 6H). ^13^C NMR (400 MHz, CDCl_3_, δ): 186.04, 159.02, 155.75, 153.57, 147.57, 145.34, 137.65, 136.64, 135.73, 134.53, 134.04, 133.87, 130.36, 120.06, 115.12, 115.02, 114.80, 114.57, 113.59, 112.55, 112.36, 68.75, 55.67, 40.40, 40.07, 34.75, 33.62, 31.87, 31.83, 29.96, 29.63, 29.57, 29.31, 27.64, 26.61, 23.27, 22.81, 22.65, 14.08, 13.73, 10.26. MS (MALDI-TOF) m/z: calcd 1590.70, found M^+^: 1590.60.

*Compound T-OD:*

The starting material compound 3-T should be compound OD (128 mg, 0.1 mmol). The final product compound T-OD was obtained as a dark solid (136 mg, 80 %).^1^H NMR (400 MHz, CDCl_3_, δ): 9.13 (s, 2H), 8.57-8.53 (q, 2H), 7.71-7.68 (t, 2H), 4.80-4.79 (d, 4H), 3.13-3.15 (d, 4H), 2.12-2.08 (m, 4H), 1.40-1.30 (m, 68H), 1.09-1.00 (m, 12H), 0.86-0.76 (m, 12H), 0.78-0.76 (t, 6H), 0.68-0.65 (t, 6H). ^13^C NMR (400 MHz, CDCl_3_, δ): 186.03, 159.00, 155.74, 153.58, 147.57, 145.33, 137.65, 136.64, 135.74, 134.52, 134.04, 133.86, 130.70, 120.04, 115.13, 115.03, 114.81, 114.56, 113.59, 112.54, 112.36, 68.75, 55.67, 40.40, 40.05, 34.73, 33.60, 31.91, 31.88, 29.97, 29.68, 29.64, 29.58, 29.36, 29.32, 27.64, 26.62, 23.27, 22.82, 22.67, 14.09, 13.74, 10.26. MS (MALDI-TOF) m/z: calcd 1702.82, found M^+^: 1702.656.

**Device fabrication and characterization**

*Small-area device fabrication*

The device structure used in the small-area cell fabrication was indium tin oxide (ITO)/ZnO nanoparticles/PEIE/photoactive layer/MoO_3_/Ag. The pre-patterned ITO/glass substrates were ultrasonicated in the order of detergent, acetone, and isopropanol. After being dried at around 140 °C in an oven for 4 h, the substrates were then treated by UV-ozone exposure for 15 min. Subsequently, a thin layer of ZnO NPs (30 nm) in butanol was deposited at 3000 rpm for 30 s and baked at 110 °C for 10min. Then, polyethyleneimine, 80% ethoxylated (PEIE) solution was prepared by dissolving PEIE in ethanol at weight concentration of 0.1 wt%. A layer of PEIE (5 nm) was then deposited onto the ZnO NPs at 5000 rpm for 40 s and then annealed at 110 °C for 10 min. The ITO/ZnO NPs/PEIE substrates were transferred to an N_2_-filled glovebox for deposition of the photoactive layer. The PM6:Y6 derivatives (1.0:1.2 w/w) solutions were spin cast on top of the PEIE layer at 2500 rpm from an *o*-xylene solvent only. Afterwards, the films were annealed at 130 °C for 10 min. Finally, MoO_3_ (10 nm) and Ag (100 nm) were deposited on top of the photoactive layer in a high vacuum chamber (~10^−6^ torr). The photovoltaic properties of small area OSCs were evaluated through a photomask with an aperture size of 0.09 cm^2^_._

*Large-area OSCs module fabrication*

The modules were fabricated with an inverted architecture (ITO/ZnO nanoparticles/ photoactive layer (thickness ≈ 130 nm)/MoO_3_/Ag). For the fabrication of large-area modules, a D-bar coater, manufactured by Printed Electro Mechanical System (PEMS, South Korea), was used. First, ZnO (nanoparticles) was bar-coated with a bar speed of 10 mm s^−1^, and subsequently annealed at 150˚C for 10 min. The photoactive materials were dissolved into *o*-xylene (total concentration: ~16 mg mL^−1^) and stirred for 6 h at 70℃. Then, the photoactive layer was bar-coated (coating speed: 18 mm s^−1^) and annealed at 120˚C for 10 min. Finally, 10 nm of MoO_3_ and 100 nm of Ag were thermally evaporated to complete the module fabrication. A shadow mask having 55 cm^2^ area was used to measure the PCE of the modules.

*Efficiency and external quantum efficiency (EQE) of OSCs*

OSCs were characterized from *J–V* curve measurement using Keithley 2400 source meter and a solar simulator (K201 LAB55, McScience), under simulated 100 mW cm^−2^ irradiation from a Xe arc lamp with an AM 1.5G filter. Simulator irradiance was characterized using a calibrated spectrometer and the illumination intensity was set using an NREL-certified silicon diode with an integrated KGI optical filter. EQEs were measured using a spectral measurement system (K3100 IQX, McScience Inc.) which applied monochromatic light from the 100 W Xe arc lamp filtered by an optical chopper and a monochromator.

*Hole- and electron-only device fabrication and carrier mobility measurements*

By employing the space-charge-limited current (SCLC) method, charge carrier mobilities were measured which is expressed by the equation as *J* = 9ε_0_ε_r_μ*V*^2^/8*L*^3^. Here, *L* is the thickness of the photoactive layer, *J* is the current density, μ is the electron/hole mobility, ε_0_ is the permittivity of free space (8.85 × 10^−12^ F m^−1^), ε_r_ is the relative dielectric constant of the transport medium, and V is the internal voltage in the device (*V*= *V*_appl_‒*V*_bi_‒*V*_r_). *V*_appl_ is the applied voltage to the device, *V*_bi_ is the built-in voltage, and *V*_r_ is the voltage drop due to contact resistance and series resistance across the electrode. Hole-only devices were fabricated as ITO/PEDOT:PSS/photoactive films/Au, while electron only devices were fabricated with a structure of ITO/ZnO NPs/PEIE/photoactive films/Ca/Al.

*Grazing-incidence wide-angle X-ray scattering (GIWAXS) analysis*

GIWAXS measurements were performed at 3C beamline of the Pohang Accelerator Laboratory (PAL) in Republic of Korea. The X-ray radiation beam energy was 11.07 keV (wavelength: 1.11996 Å) with a sample-to-detector distance of approximately 222 mm. The incidence angle for the X-ray beam was set to 0.12 to characterize the optimized neat and blend films. The GIWAXS patterns were collected from a 2D CCD detector (Rayonix SX165). The measured data were analyzed using the Igor-Pro software package. The GIWAXS samples were prepared by employing Si-wafer/ZnO NPs/PEIE/thin films as fabricated in the optimized device condition.

*Atomic force microscopy (AFM)*

AFM samples were prepared by using the optimal conditions but without the MoO_3_/Ag electrode.


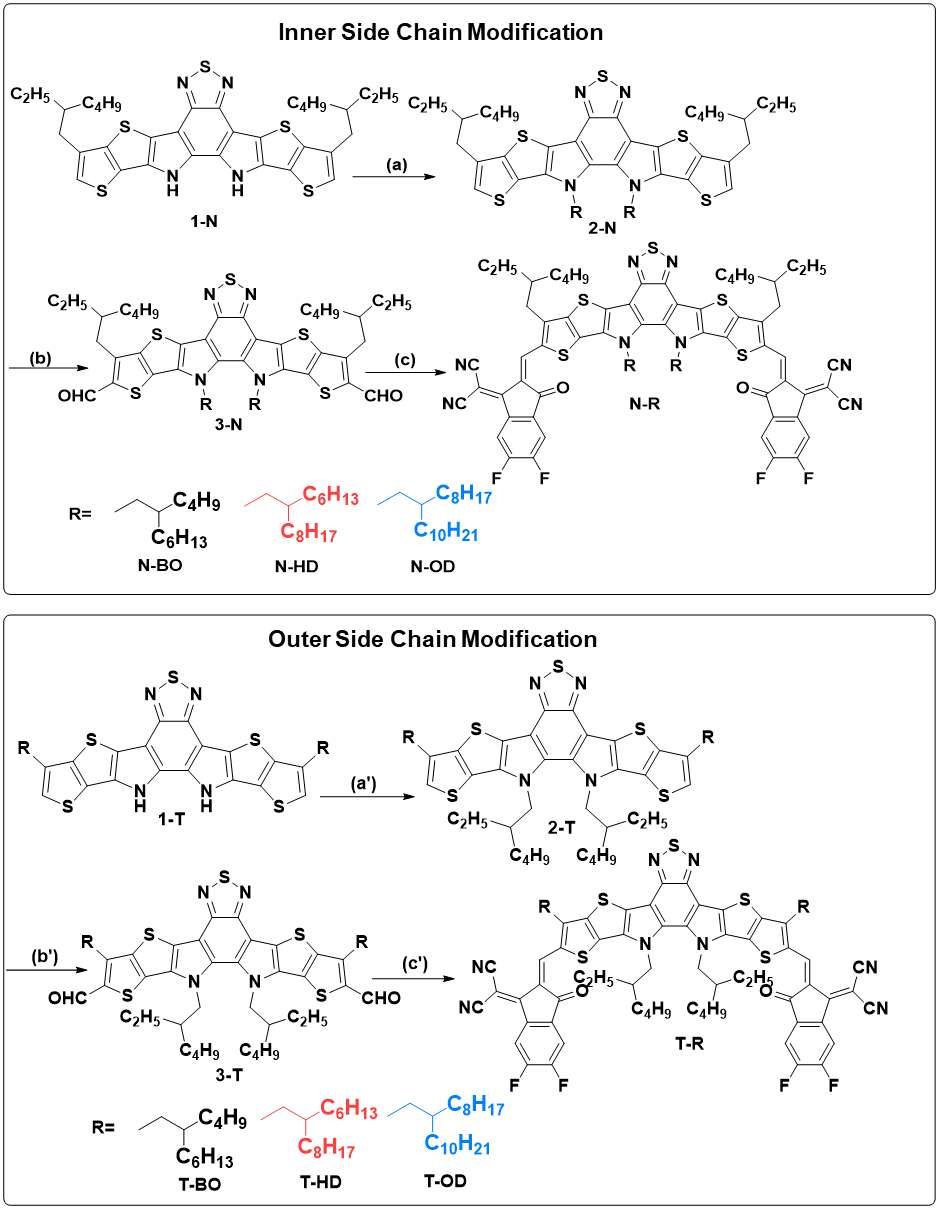


**Figure S1.** Synthetic routes towards branched chain modification N-R and T-R series. Reagents and conditions (a) (2-N) K_2_CO_3_, KI, DMF, R-Br, 90 °C; (a’)(2-T) K_2_CO_3_, KI, DMF, 1-bromo-2-ethylhexane, 90 °C (b)(b’) POCl_3_, DMF, ClCH_2_CH_2_Cl, 70 °C; (c)(c’) 2-(5,6-difluoro-3-oxo-2,3-dihydro-1H-inden-1-ylidene)malononitrile, Pyridine, CHCl_3_, 65 °C.


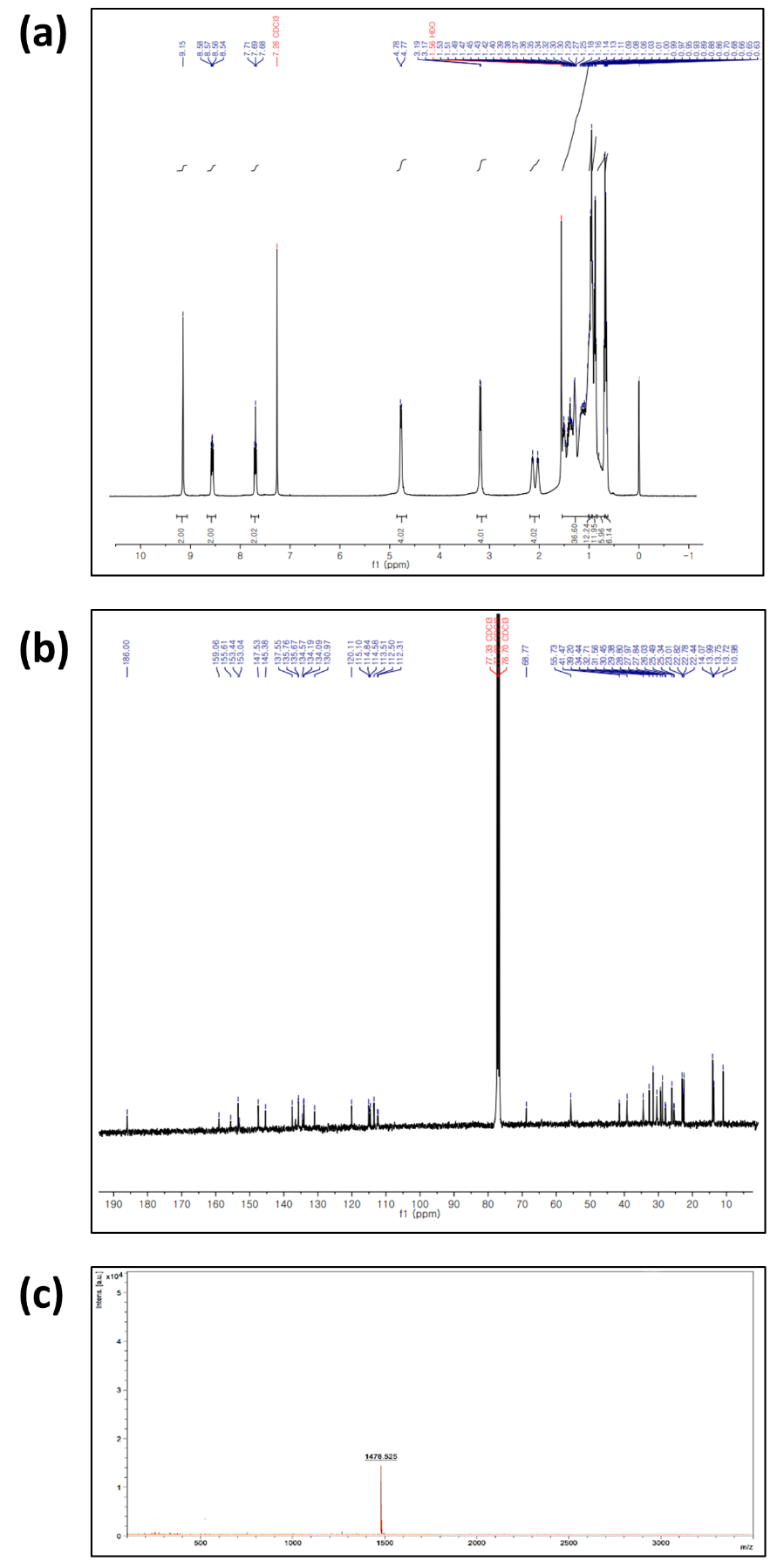


**Figure S2.** a) ^1^H NMR spectrum, b) ^13^C NMR spectrum, and c) MS (MALDI-TOF) spectrum of N-BO.


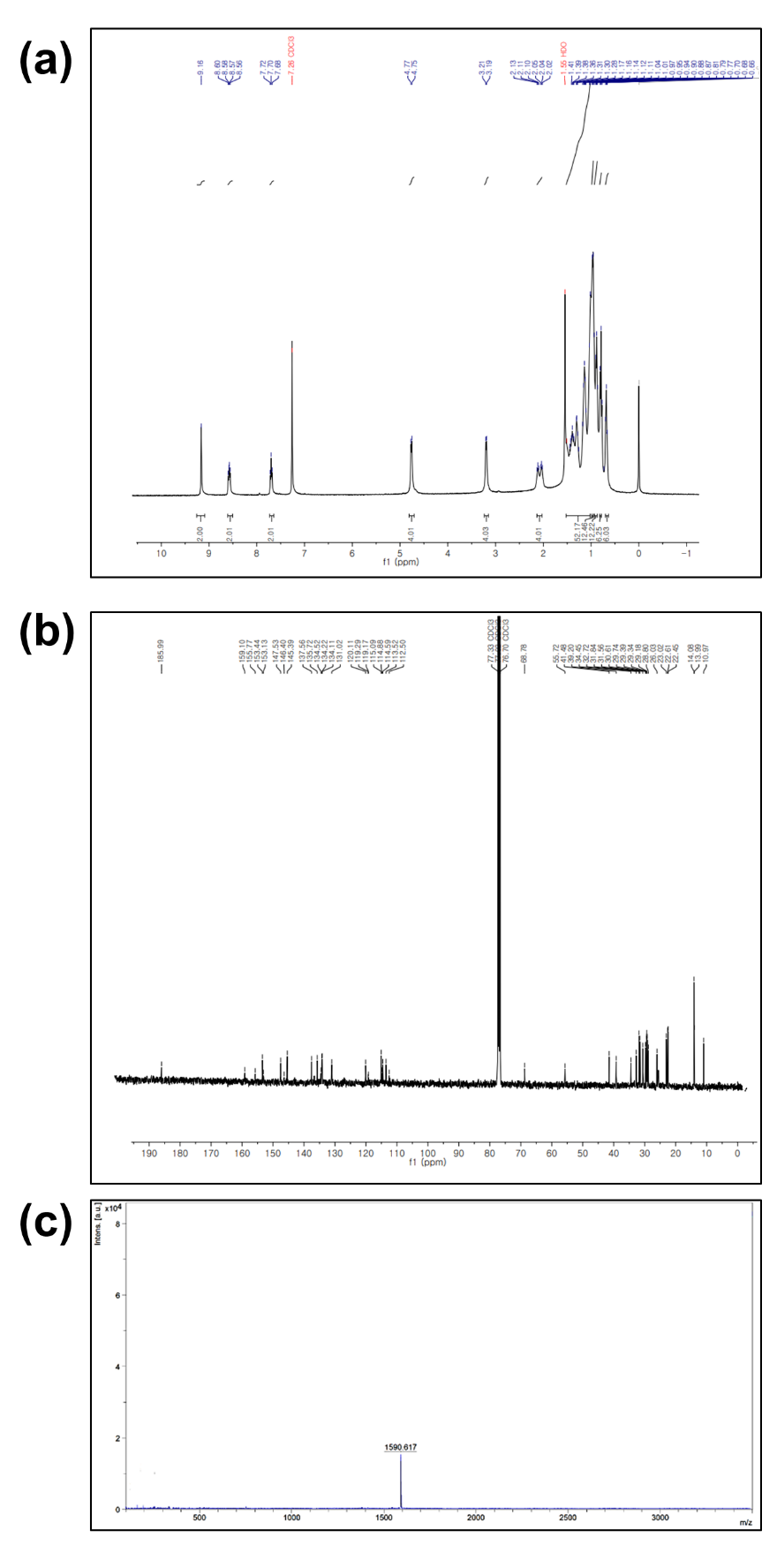


**Figure S3.** a) ^1^H NMR spectrum, b) ^13^C NMR spectrum, and c) MS (MALDI-TOF) spectrum of N-HD.


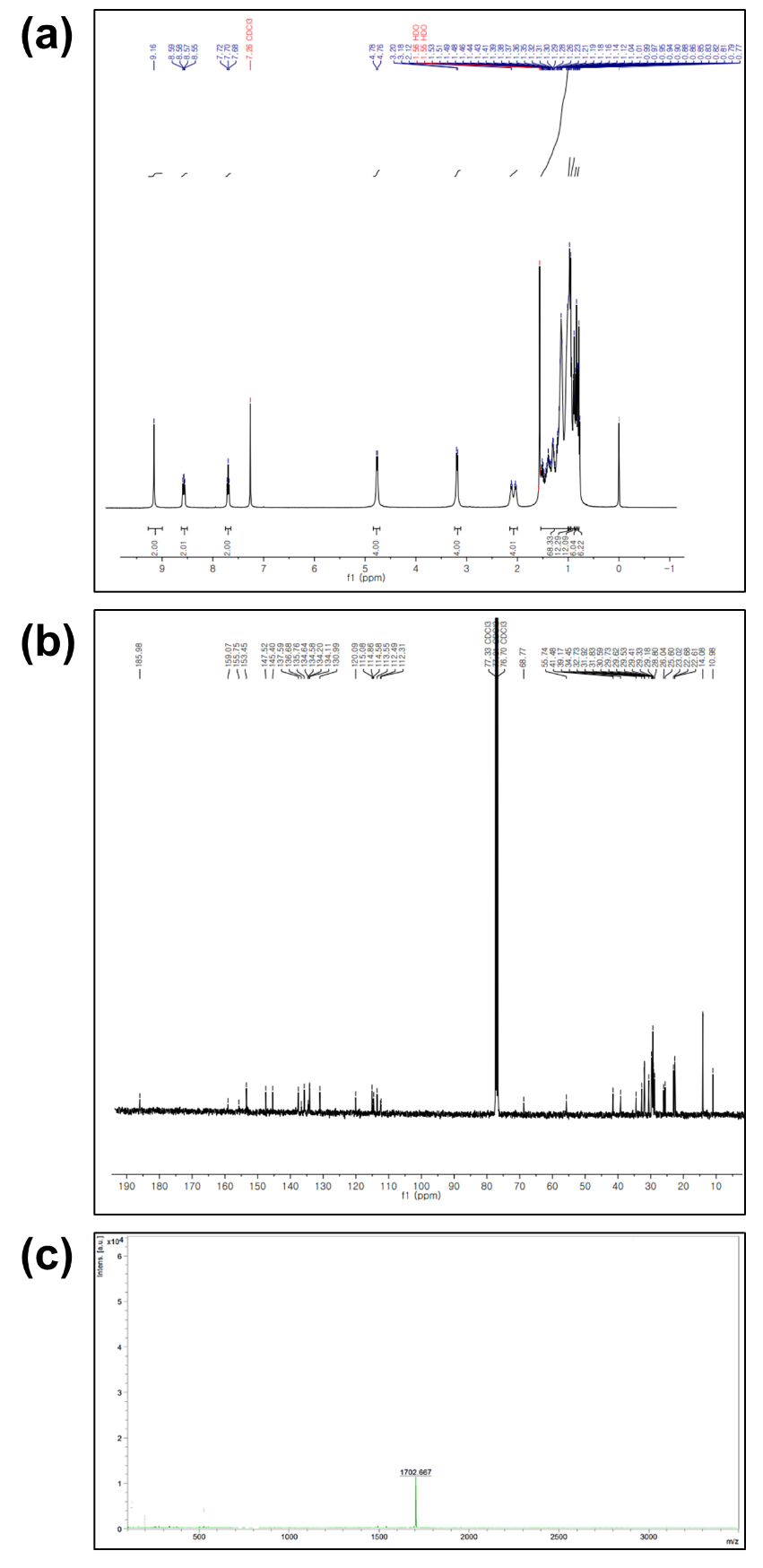


**Figure S4.** a) ^1^H NMR spectrum, b) ^13^C NMR spectrum, and c) MS (MALDI-TOF) spectrum of N-OD.


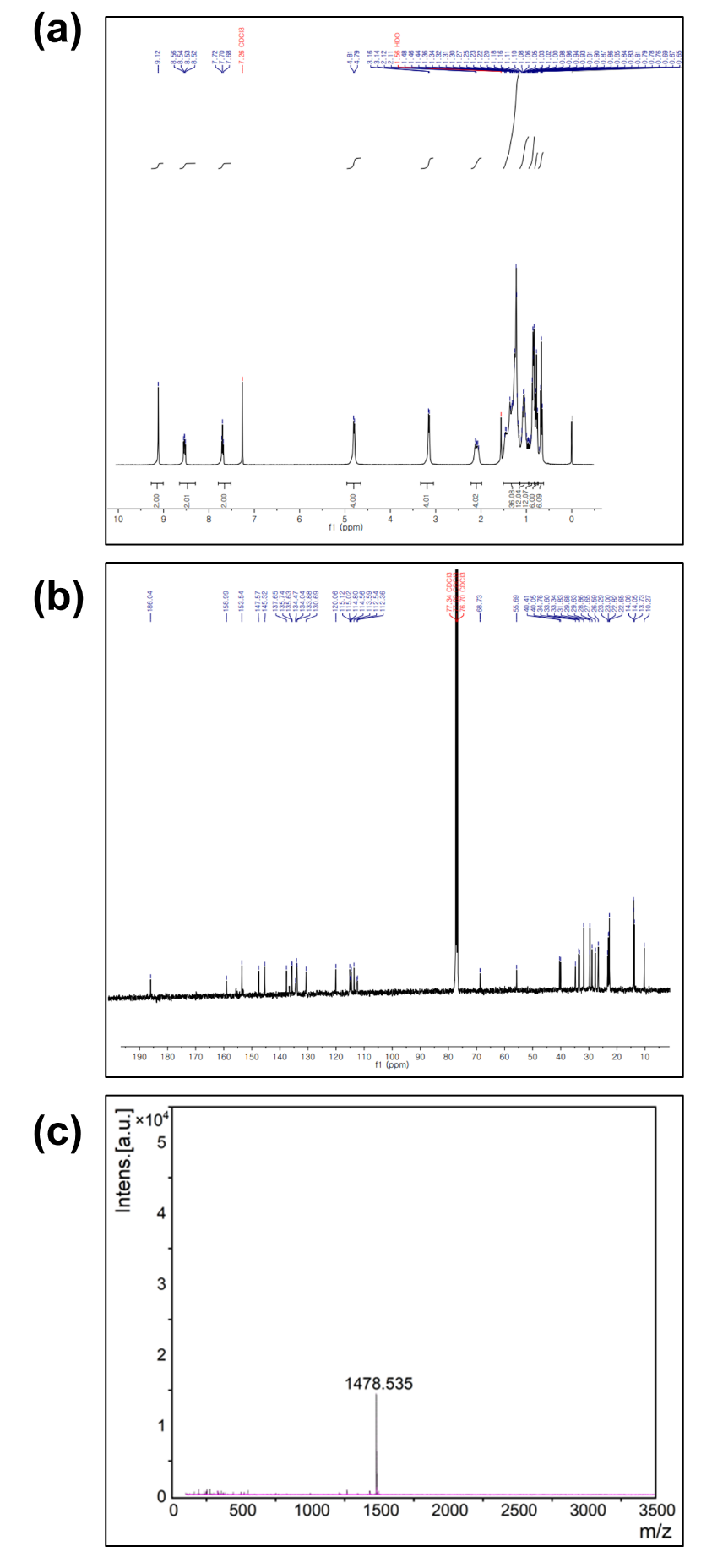


**Figure S5.** a) ^1^H NMR spectrum, b) ^13^C NMR spectrum, and c) MS (MALDI-TOF) spectrum of T-BO.


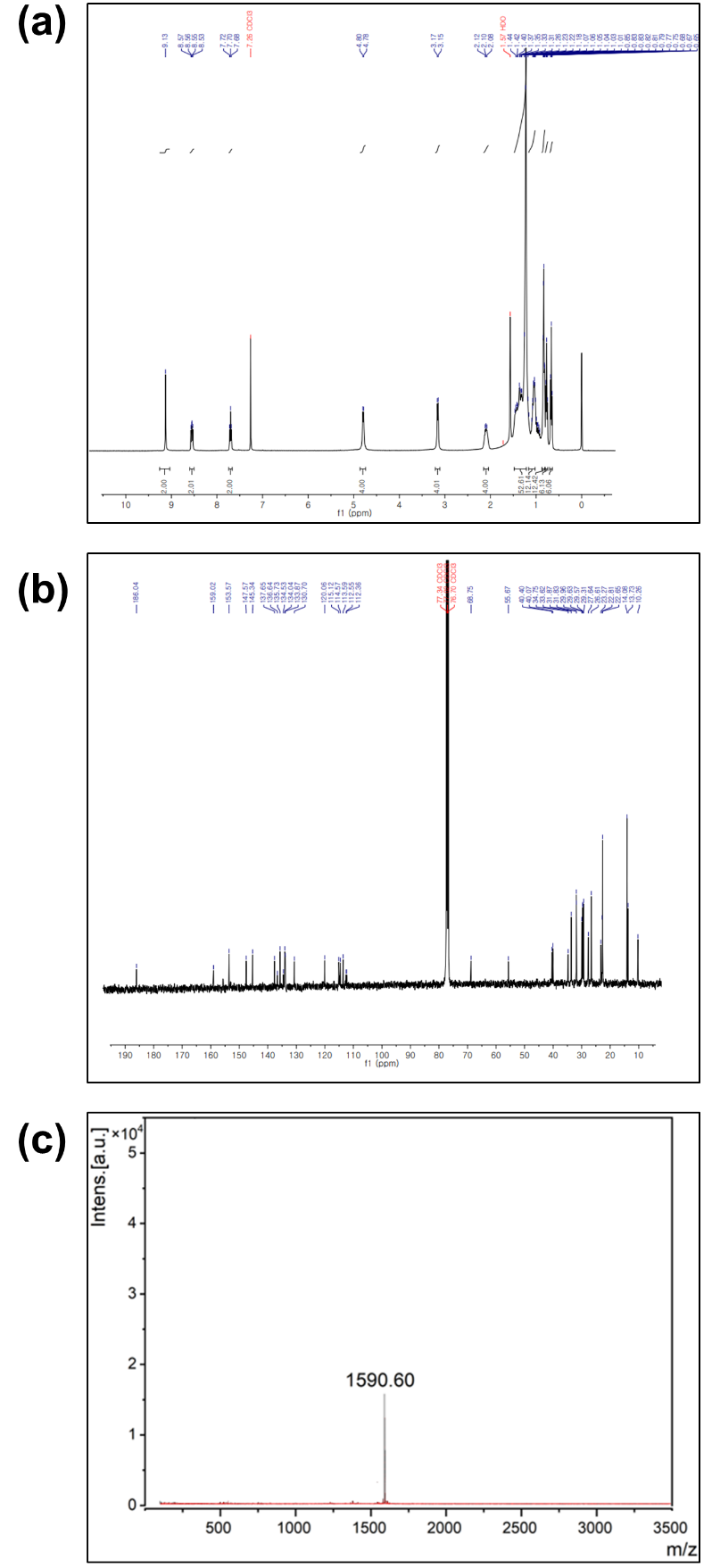


**Figure S6.** a) ^1^H NMR spectrum, b) ^13^C NMR spectrum, and c) MS (MALDI-TOF) spectrum of T-HD.


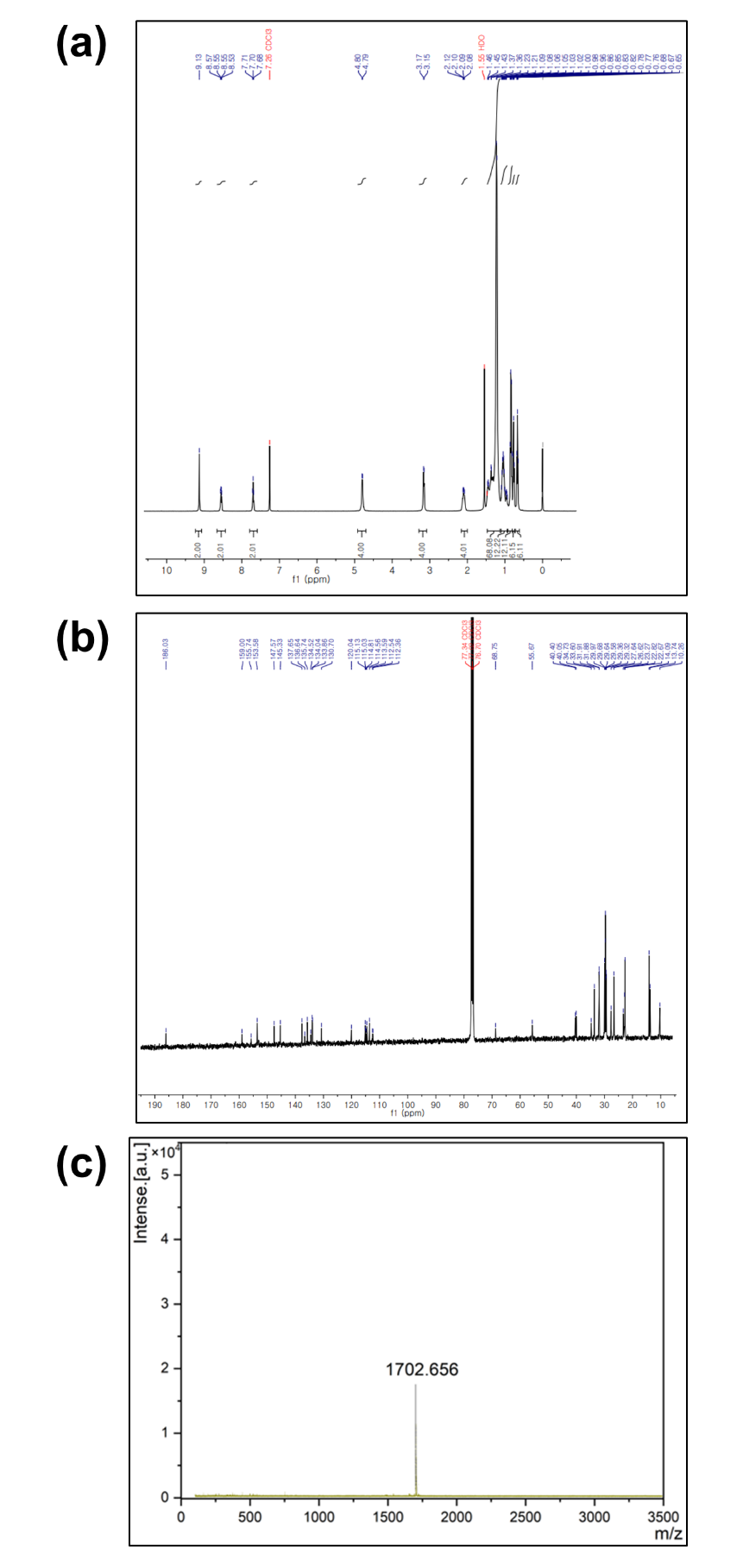


**Figure S7.** a) ^1^H NMR spectrum, b) ^13^C NMR spectrum, and c) MS (MALDI-TOF) spectrum of T-OD.

**Figure S8.** Thermogravimetric analysis (TGA) traces of NFAs.


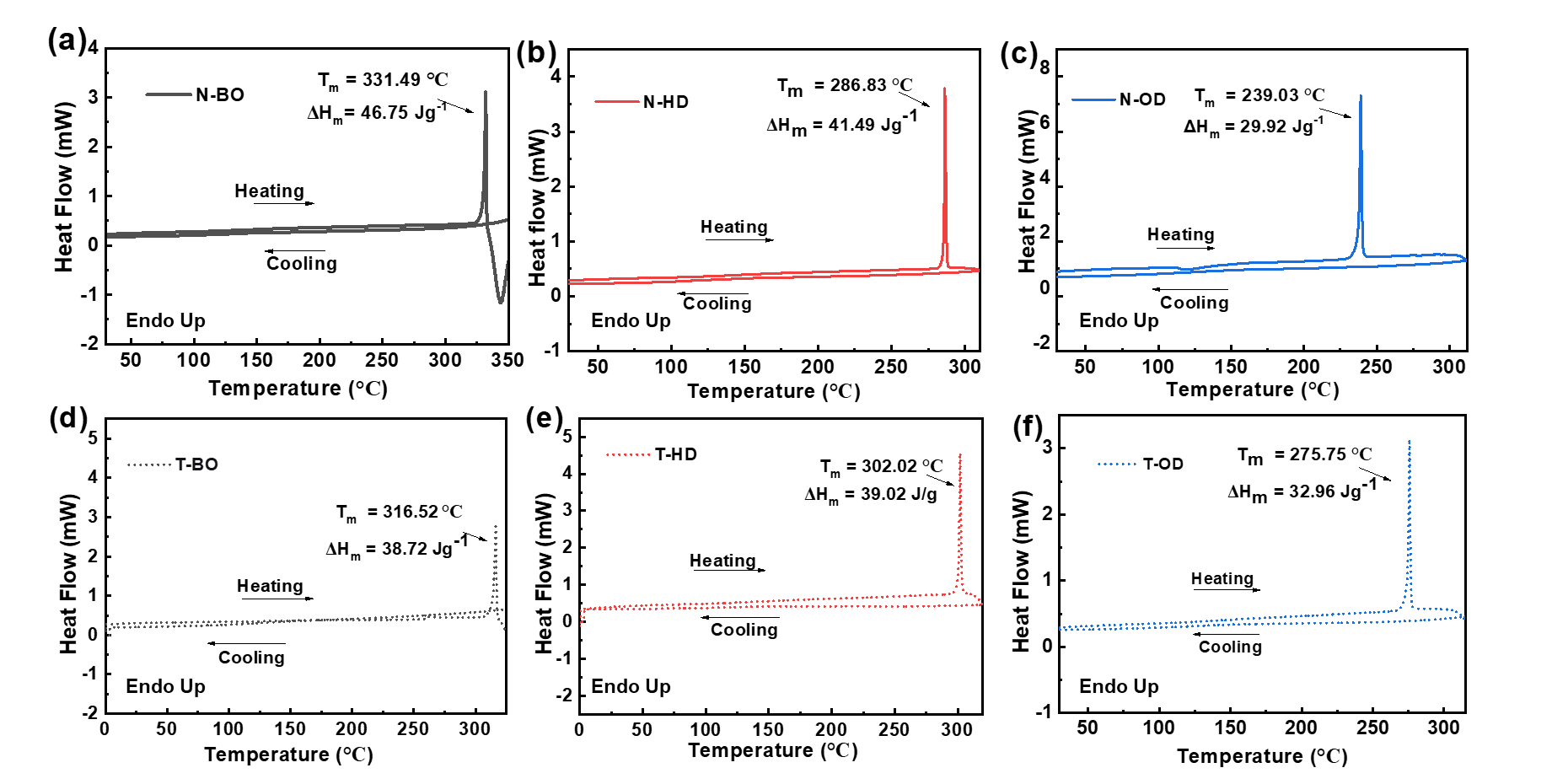


**Figure S9.** a-f) Differential scanning calorimetry (DSC) thermograms for NFAs.

**Table S1**. Solubility of NFAs in *o*-xylene at room temperature.

| **NFA** | **Solubility [mg mL^−1^]** |
| --- | --- |
| **N-BO** | 15 |
| **N-HD** | 113 |
| **N-OD** | 134 |
| **T-BO** | 18 |
| **T-HD** | 110 |
| **T-OD** | 132 |


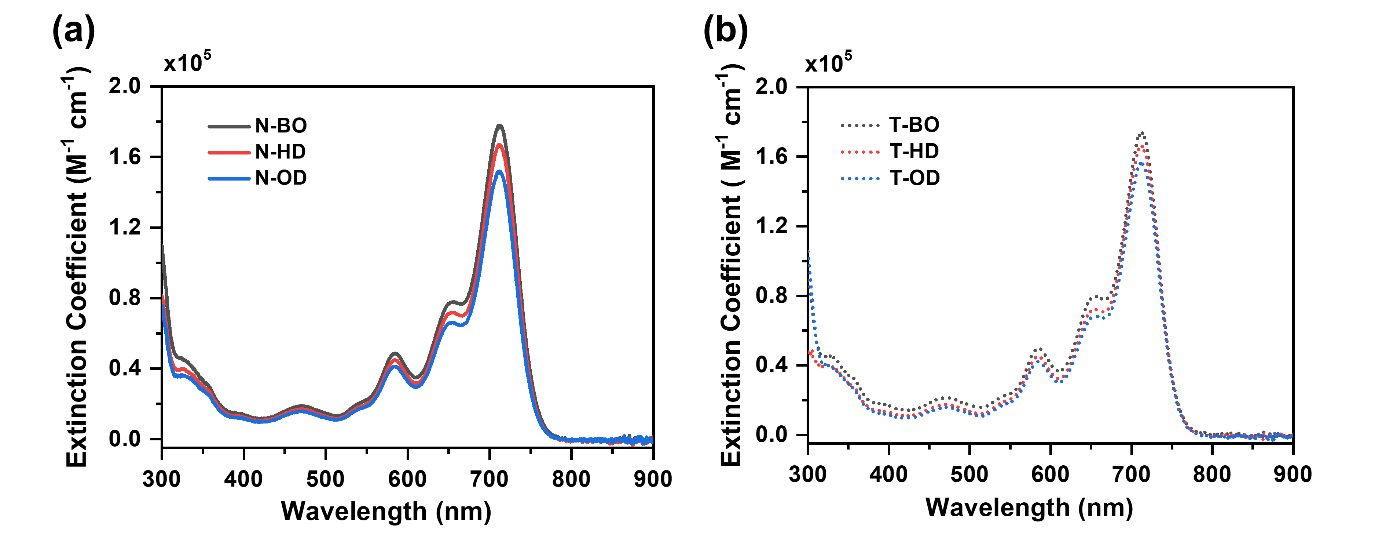


**Figure S10.** Absorption coefficient spectra of a) N-series NFAs and b) T-series NFAs in dilute *o*-xylene solution (1.0 × 10^−5^ M).


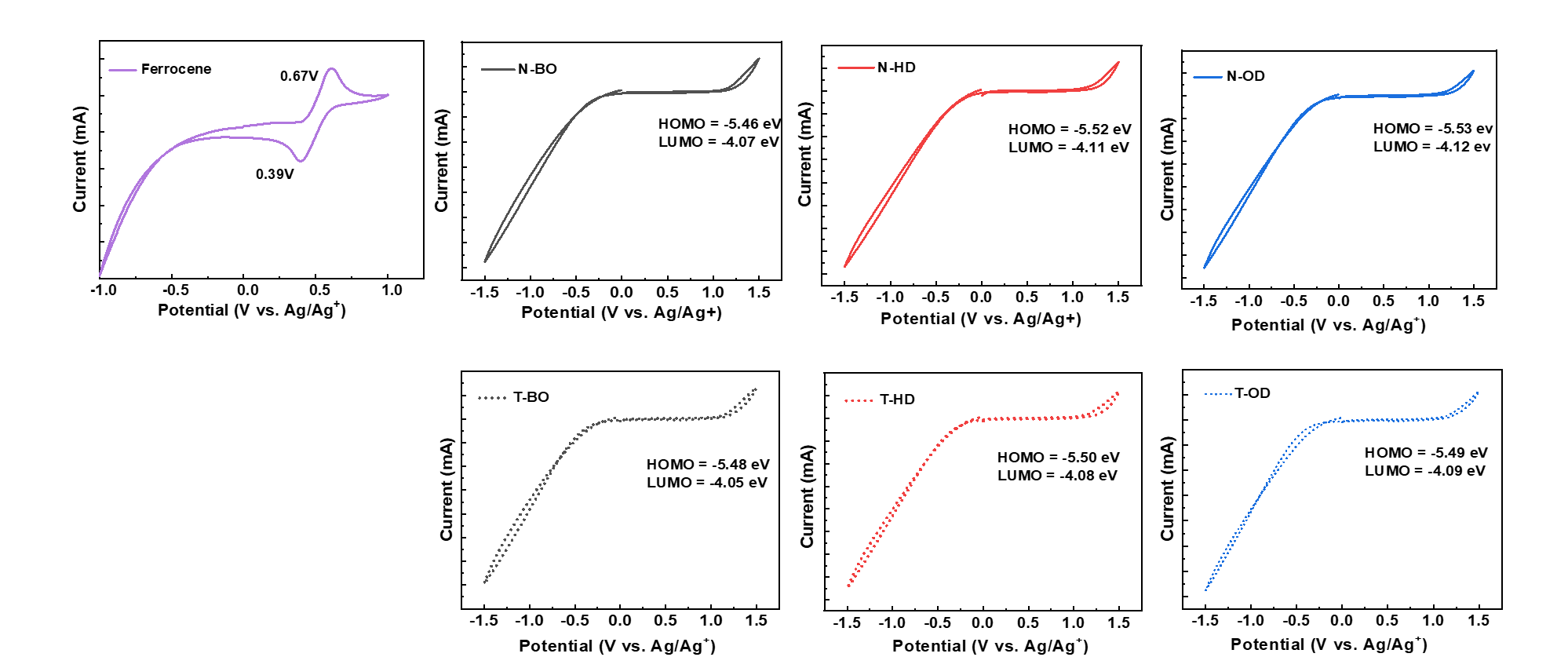


**Figure S11.** CV traces of NFAs.


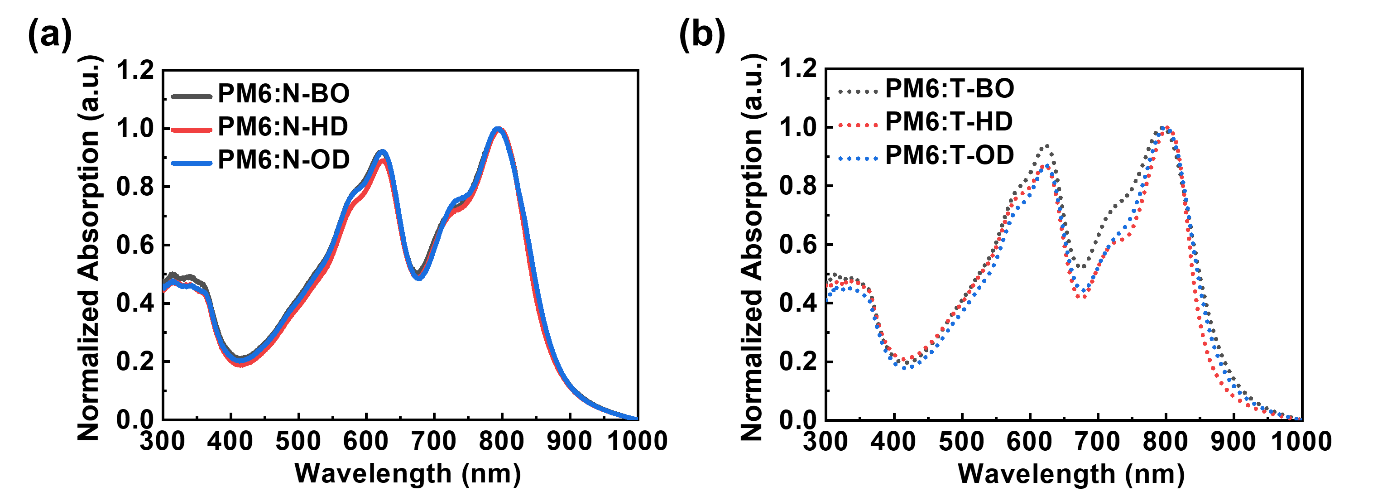


**Figure S12**. Thin-film absorption spectra of a) PM6:N-series NFAs and b) PM6:T-series NFAs blend films.


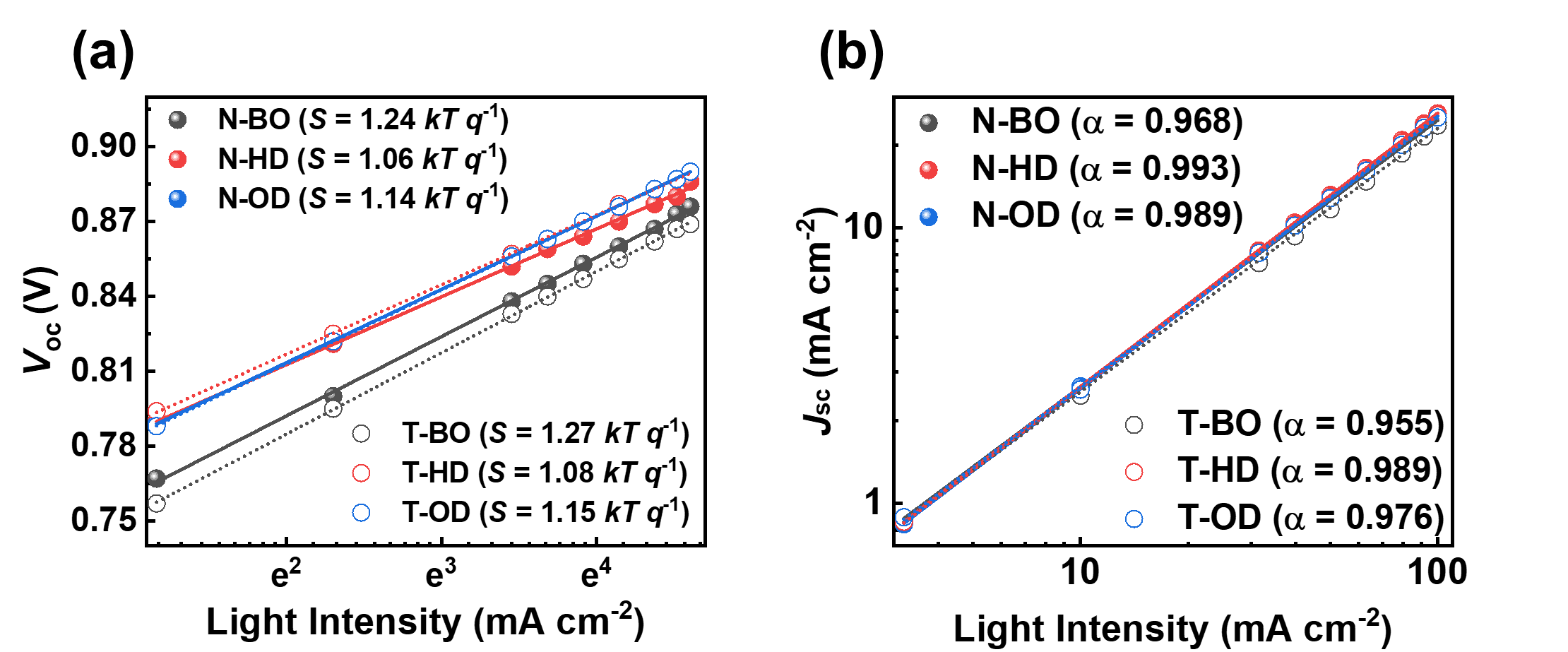


**Figure S13.** Light intensity (*P*_light_)-dependent a) *V*oc and b) *J*_SC_ characteristics of PM6:NFA OSCs.


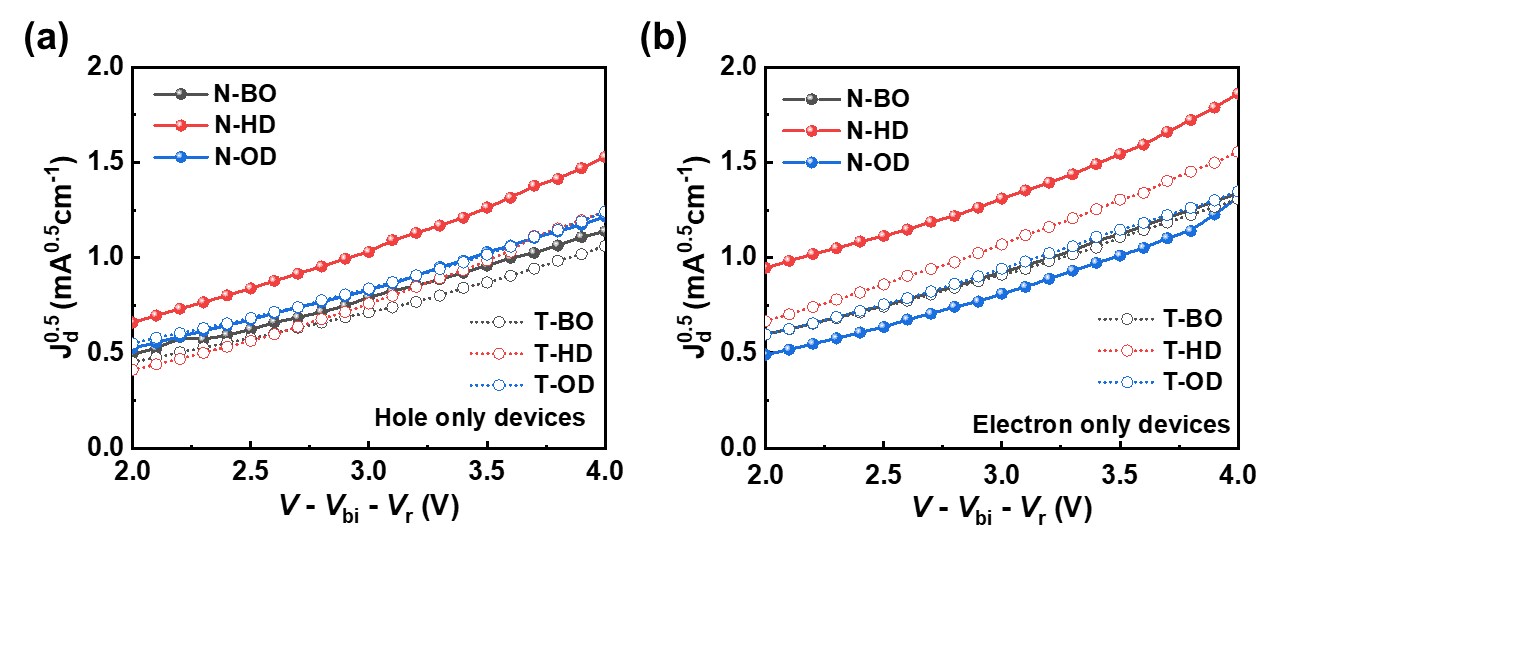


**Figure S14.** *J*_d_^0.5^ vs. *V*−*V*_bi_−*V*_r_ curves of a) hole-only and b) electron-only devices of PM6:NFA blend films.


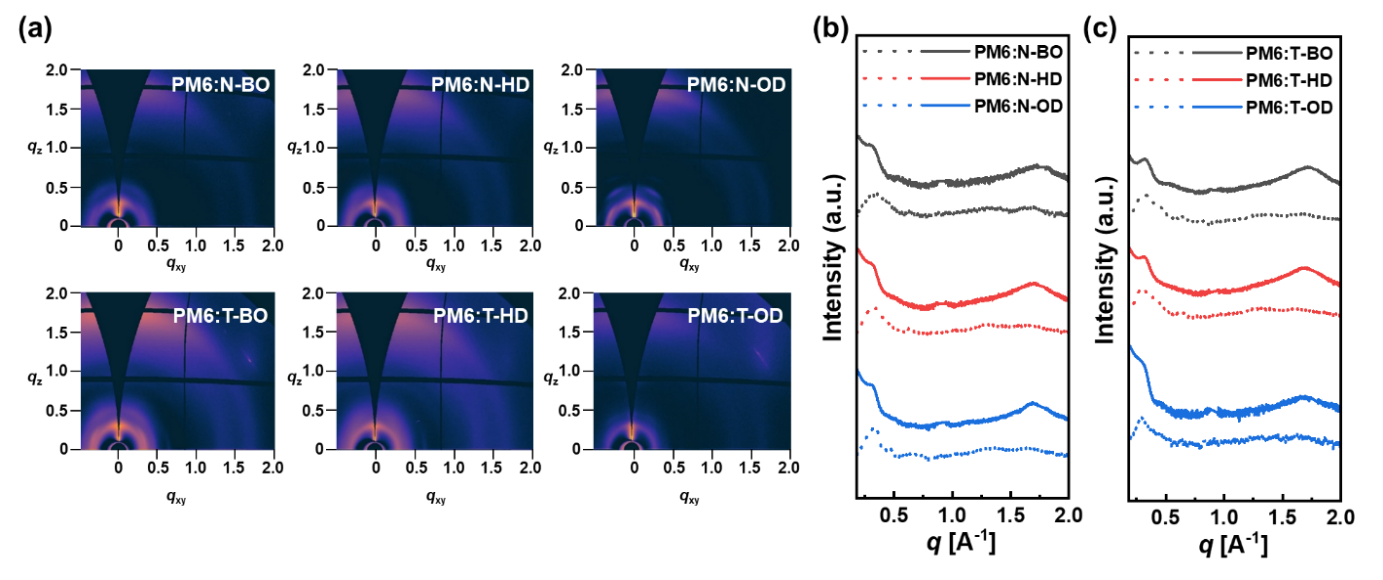


**Figure S15.** a) 2D-GIWAXS pattern images of N and T-series blend films; Line-cut profiles of b) PM6:N-series and c) PM6:T-series blend films along the IP (dotted lines) and OOP (solid lines) directions.

**Table S2.** Crystallographic parameters of PM6:NFA blend films.

| **Blend Film** | **In-plane (IP)** | | **Out-of-plane (OOP)** | | |
| --- | --- | --- | --- | --- | --- |
|  | ***q*_100_**  **[Å^−1^]** | ***d*_100_**  **[Å]** | ***q*_010_**  **[Å^−1^]** | ***d*_π–π_**  **[Å]** |  |
| **PM6:N-BO** | 0.337 | 18.6 | 1.723 | 3.647 |  |
| **PM6:N-HD** | 0.321 | 19.6 | 1.690 | 3.718 |  |
| **PM6:N-OD** | 0.315 | 19.9 | 1.678 | 3.744 |  |
| **PM6:T-BO** | 0.323 | 19.5 | 1.710 | 3.674 |  |
| **PM6:T-HD** | 0.303 | 20.7 | 1.687 | 3.724 |  |
| **PM6:T-OD** | 0.296 | 21.2 | 1.666 | 3.771 |  |


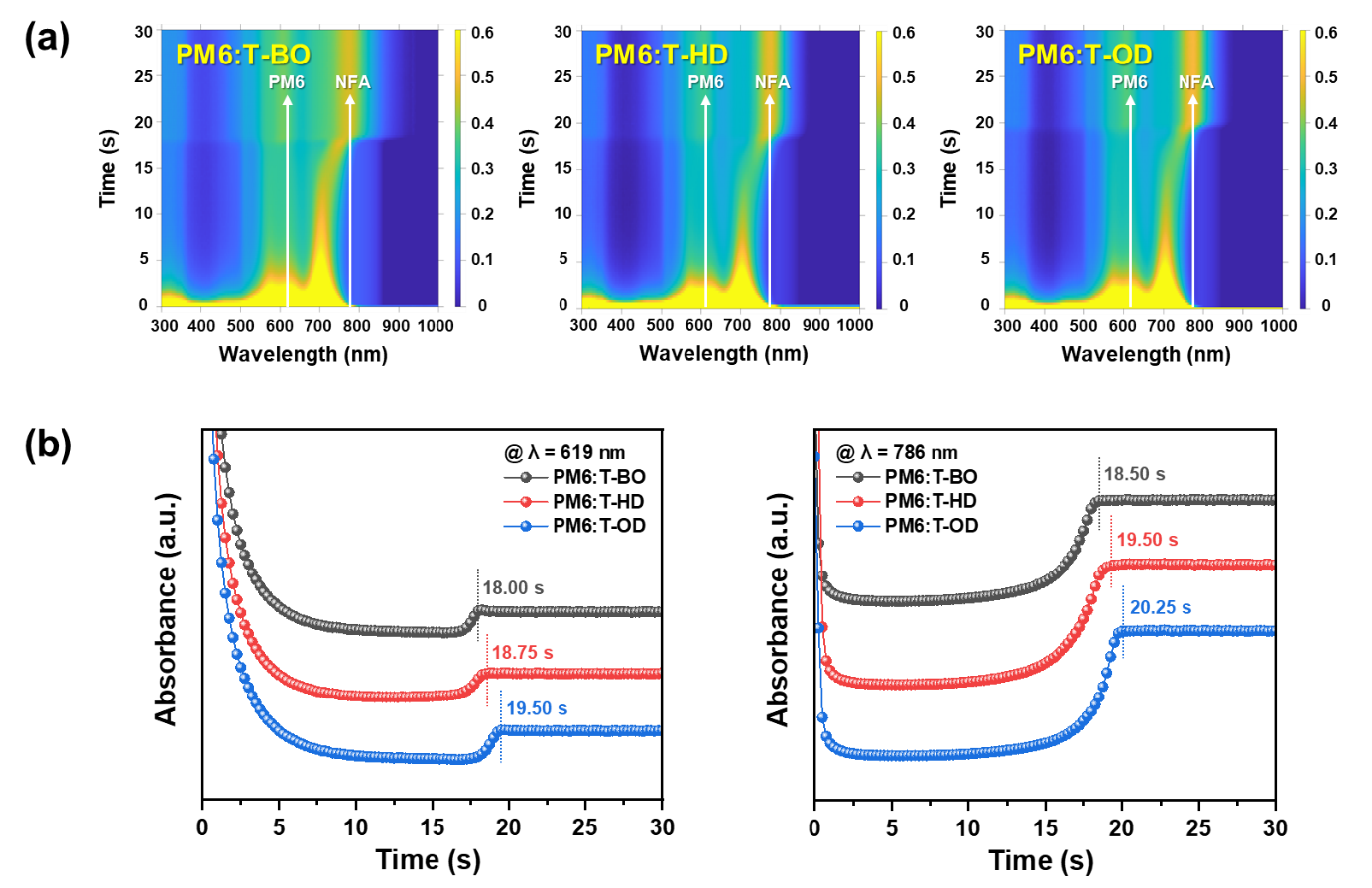


**Figure S16.** a) 2D contour maps of PM6:T-BO, PM6:T-HD, and PM6:T-OD blend films obtained by in situ UV–Vis spectroscopy and their b) line-cut profiles at λ = 619 and 786 nm.


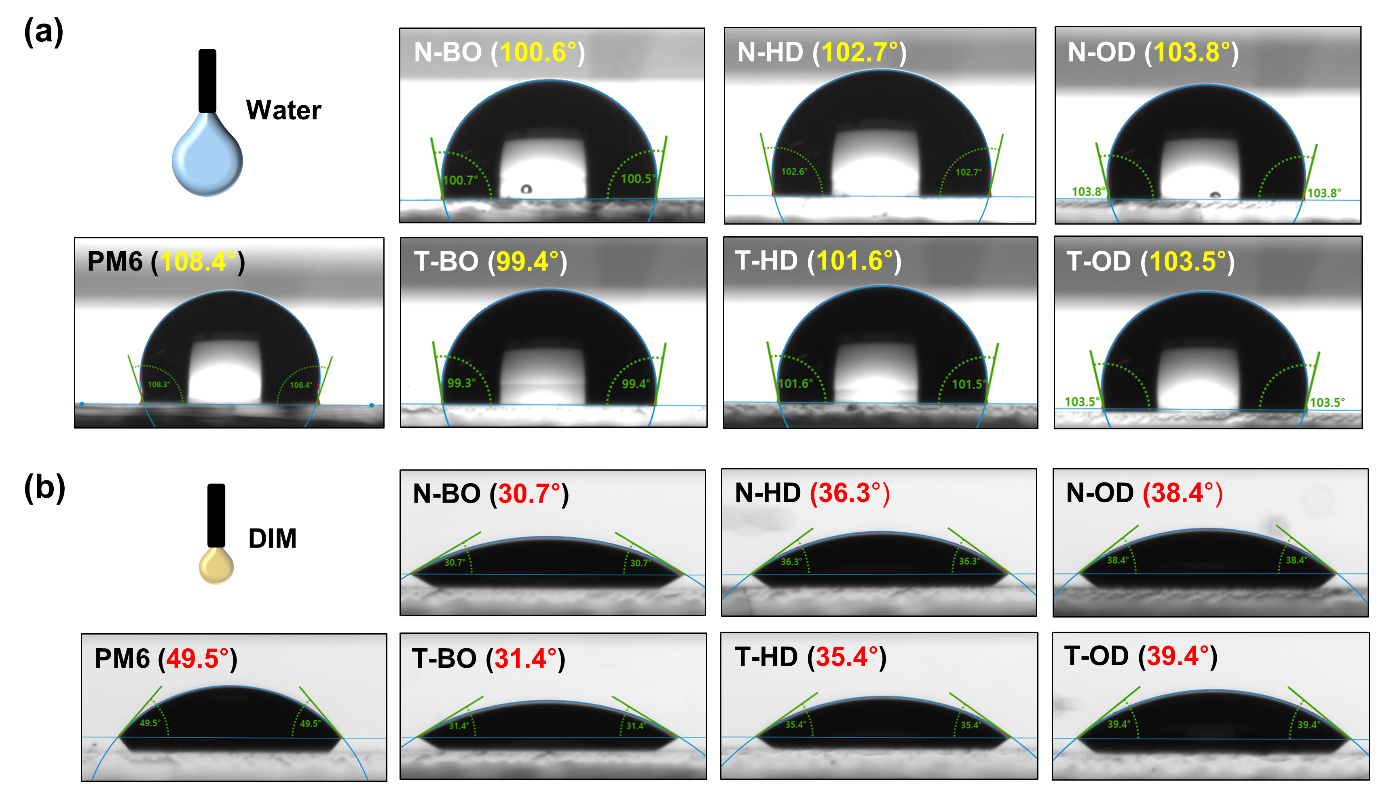


**Figure S17.** Contact angles of PM6 and NFA neat films with (a) water and (b) diiodomethane (DIM).

**Table S3.** Contact angles of water and diiodomethane (DIM) droplets with each film, and surface tensions and interfacial tensions of the materials calculated based on the contact angles.

| **Material** | **Contact Angle** | | **Surface Tension**  **[mN m^−1^]** | ***γ*_PM6–NFA_ [mN m^−1^]^a^** |
| --- | --- | --- | --- | --- |
|  | ***θ*_water_ [°]** | ***θ*_DIM_ [°]** |  |  |
| **PM6** | 108.4 | 49.5 | 36.24 | - |
| **N-BO** | 100.6 | 30.7 | 44.30 | 2.21 |
| **N-HD** | 102.7 | 36.3 | 41.84 | 1.47 |
| **N-OD** | 103.8 | 38.4 | 40.88 | 1.12 |
| **T-BO** | 99.4 | 31.4 | 44.25 | 2.59 |
| **T-HD** | 101.6 | 35.4 | 42.33 | 1.83 |
| **T-OD** | 103.5 | 39.4 | 40.45 | 1.23 |

^a^Interfacial tension with PM6 donor.


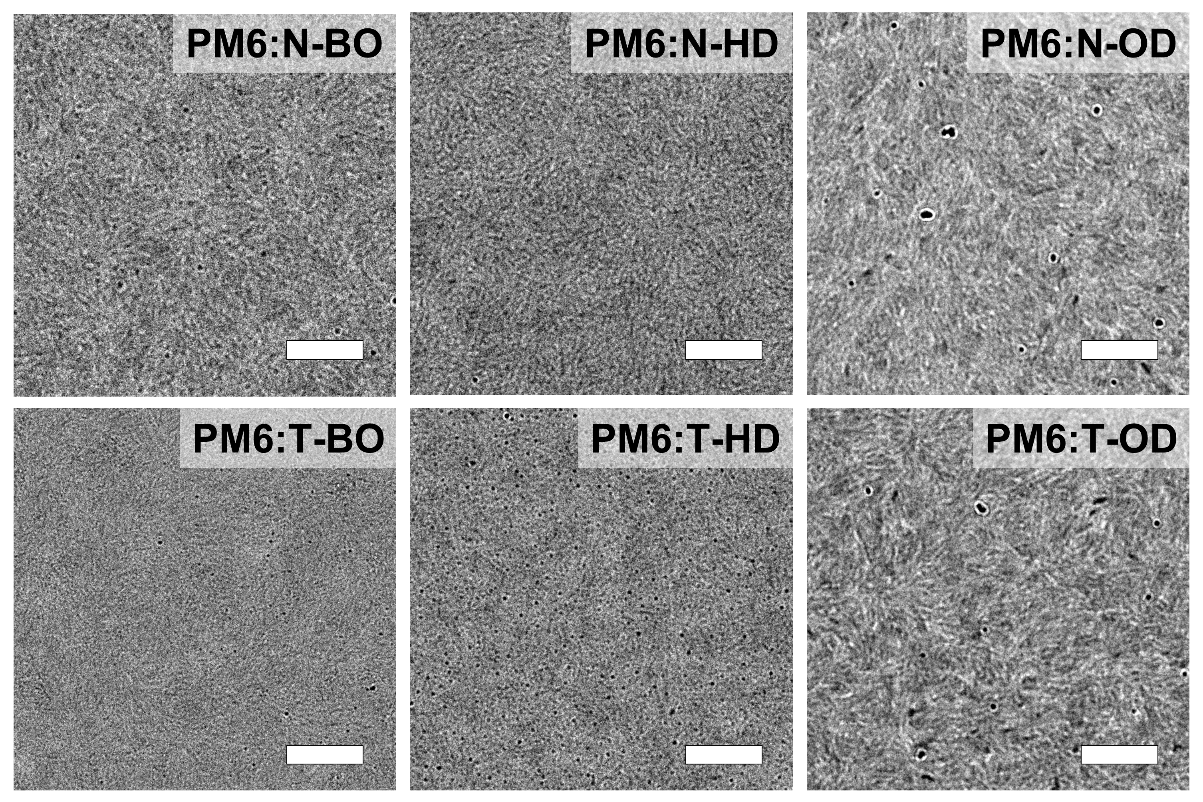


**Figure S18**. TEM images of PM6:NFA blend films. The scale bars indicate 300 nm.

**Table S4.** Photovoltaic performance and active area of reported sub-modules in literature processed by using halogenated or non-halogenated solvents.

| **Photoactive layer** | **Active Area [cm^2^]** | **Processing**  **Solvent** | ***V*_OC_ [V]** | ***J*_SC_ [mA cm^−2^]** | **FF [%]** | **PCE**  **[%]** | **Ref.** |
| --- | --- | --- | --- | --- | --- | --- | --- |
| PBDB-TF:IT-4F | 12.6 | CB | 2.56 | 6.23 | 64.02 | 10.21 | 2 |
| SMD2:ITIC | 80 | CB+DIO | 8.80 | 1.12 | 53.1 | 5.25 | 3 |
| TPD-3F:IT-4F | 20.4 | CB | 4.52 | 71.4^a^ | 64.0 | 10.13 | 4 |
| NF3000-P: NF3000-N | 10.8 | CB | 2.76 | 5.29 | 67.0 | 9.80 | 5 |
| PM6:Y6 | 11.52 | CF | 3.20 | 6.41 | 57.85 | 11.86 | 6 |
| PBDB-T:ITIC | 58.5 | CB | 8.801 | 1.71 | 60.0 | 9.03 | 7 |
| PBDB-T:ITIC | 52 | CB | 0.86 | 8.0 | 0.32 | 2.2 | 8 |
| PM6:BTP-4CL-12 | 30 | CB | 6.2 | 2.9 | 48.9 | 8.8 | 9 |
| P3HT:IDTBR | 59.52 | CB | 8.388 | 9.76 | 64.9 | 4.4 | 10 |
| BDT-Th10 | 58.5 | CB | 7.527 | 1.55 | 66.34 | 7.74 | 11 |
| PTB7-Th:PC_70_BM | 16.60 | CB | 2.37 | 4.87 | 58 | 7.4 | 12 |
| P3HT:PCBM | 10.8 | CF+CB | 0.62 | 10.4 | 0.54 | 3.5 | 13 |
| PM6:DTY6 | 18 | *o*-xylene | 5.11 | 3.89 | 72.5 | 14.4 | 14 |
| PM6:Y6 | 36 | *p*-xylene | 9.04 | 1.63 | 49.42 | 7.31 | 15 |
| PNTz4T-5MTC:PC_71_BM | 54.45 | *o*-xylene | 7.970 | 1.3406 | 61 | 6.61 | 16 |
| PBDB-T:CNDTBT-C8IDT-FINCN | 55.45 | CB | 8.16 | 1.68 | 67.2 | 9.21 | 17 |
| PTB7-Th:EH-IDTBR | 55.5 | toluene | 11.47 | 1.29 | 63 | 9.32 | 18 |
| PBDB-T:CNDTBT-IDTT-FINCN | 55.45 | CB | 9.37 | 1.25 | 64 | 7.5 | 19 |
| PTB7-Th:PC71BM | 16 | 2-methylanisole | 3.04 | 4.5 | 55 | 7.5 | 20 |
| PTB7-Th:PC_71_BM | 93 | CB | 8.91 | 1.2 | 42 | 4.49 | 21 |
| PM6:Y6-HU | 31.50 | *o*-xylene | 4.27 | 4.65 | 62.61 | 12.44 | 22 |
| POD2T-DTBT:PC_71_BM | 24 | DCB | 5.66 | 1.20 | 0.580 | 3.93 | 23 |
| PDTBT-*alt*-TT:PC_71_BM | 24 | DCB | 6.10 | 1.45 | 0.631 | 5.58 | 23 |
| PCDTBT: PC_70_BM | 33.5 | CB | 4.55 | 1.82 | 39.2 | 3.25 | 24 |
| PV2000:PCBM | 23.7 | *o*-xylene | 4.036 | 67.96^a^ | 65.3 | 7.56 | 25 |
| PTB7-Th:*p*-DTS(FBTTH_2_)_2_ | 20 | *o*-xylene | 2.71 | 3.89 | 49.19 | 5.18 | 26 |
| PBTPttBD-75:BTP-eC11 | 55 | *o*-xylene | 8.91 | 1.92 | 66.72 | 11.57 | 27 |
| PTF5:Y6-BO | 54.45 | *o*-xylene | 9.32 | 1.73 | 71.9 | 11.6 | 28 |
| PM6:G-trimer | 46.2 | *o*-xylene | 6.03 | 3.20 | 68.66 | 13.25 | 29 |

^a^Short-circuit current (*I*_sc_) [mA].


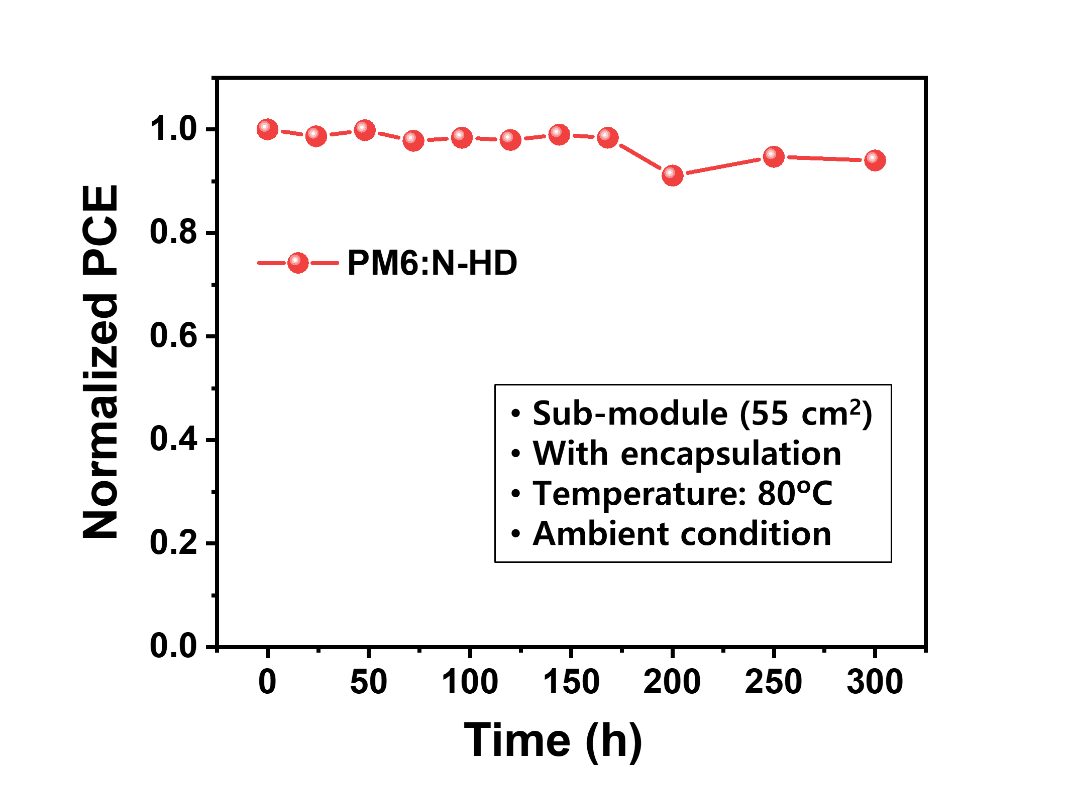


**Figure S19**. Normalized PCE of PM6:N-HD-based sub-module as a function of time stored in air at 80ºC with encapsulation.

**REFERENCES**

1. C. Li, J. Zhou, J. Song, J. Xu, H. Zhang, X. Zhang, J. Guo, L. Zhu, D. Wei, G. Han, J. Min, Y. Zhang, Z. Xie, Y. Yi, H. Yan, F. Gao, F. Liu, Y. Sun, *Nat. Energy* **2021**, *6*, 605.
2. W. Zhao, Y. Zhang, S. Zhang, S. Li, C. He, J. Hou, *J. Mater. Chem. C* **2019**, *7*, 3206.
3. Y. W. Han, S. J. Jeon, H. S. Lee, H. Park, K. S. Kim, H. W. Lee, D. K. Moon, *Adv. Energy Mater.* **2019**, *9*, 1902065.
4. C. Y. Liao, Y. Chen, C. C. Lee, G. Wang, N. W. Teng, C. H. Lee, W. L. Li, Y. K. Chen, C. H. Li, H. L. Ho, P. H. S. Tan, B. Wang, Y. C. Huang, R. M. Young, M. R. Wasielewski, T. J. Marks, Y. M. Chang, A. Facchetti, *Joule* **2020**, *4*, 189.
5. C. Y. Tsai, Y. H. Lin, Y. M. Chang, J. C. Kao, Y. C. Liang, C. C. Liu, J. Qiu, L. Wu, C. Y. Liao, H. S. Tan, Y. C. Chao, S. F. Horng, H. W. Zan, H. F. Meng, F. Li, *Sol. Energy Mater. Sol. Cells* **2020**, *218*, 110762.
6. R. Sun, Q. Wu, J. Guo, T. Wang, Y. Wu, B. Qiu, Z. Luo, W. Yang, Z. Hu, J. Guo, M. Shi, C. Yang, F. Huang, Y. Li, J. Min, *Joule* **2020**, *4*, 407.
7. S. H. Park, S. Park, S. Lee, J. Kim, H. Ahn, B. J. Kim, B. Chae, H. J. Son, *Nano Energy* **2020**, *77*, 105147.
8. L. Mao, L. Sun, B. Luo, Y. Jiang, Y. Zhou, *J. Mater. Chem. A* **2018**, *6*, 5817.
9. Y. W. Han, H. S. Lee, D. K. Moon, *ACS Appl. Mater. Interfaces* **2021**, *13*, 19085.
10. S. Strohm, F. Machui, S. Langner, P. Kubis, N. Gasparini, M. Salvador, I. McCulloch, H. J. Egelhaaf, C. J. Brabec, *Energy Environ. Sci.* **2018**, *11*, 2225.
11. S. H. Park, S. Park, D. Kurniawan, J. G. Son, J. H. Noh, H. Ahn, H. J. Son, *Chem. Mater.* **2020**, *32*, 3469.
12. S. Hong, H. Kang, G. Kim, S. Lee, S. Kim, J. H. Lee, J. Lee, M. Yi, J. Kim, H. Back, J. R. Kim, K. Lee, *Nat. Commun.* **2016**, *7*, 10279.
13. Y. Q. Wong, H. F. Meng, H. Y. Wong, C. S. Tan, C. Y. Wu, P. T. Tsai, C. Y. Chang, S. F. Horng, H. W. Zan, *Org. Electron.* **2017**, *43*, 196.
14. S. Dong, T. Jia, K. Zhang, J. Jing, F. Huang, *Joule* **2020**, *4*, 2004.
15. H. Chen, R. Zhang, X. Chen, G. Zeng, L. Kobera, S. Abbrent, B. Zhang, W. Chen, G. Xu, J. Oh, S. H. Kang, S. Chen, C. Yang, J. Brus, J. Hou, F. Gao, Y. Li, Y. Li, *Nat. Energy* **2021**, *6*, 1045.
16. S. Rasool, D. Van Vu, C. E. Song, H. K. Lee, S. K. Lee, J. C. Lee, S. J. Moon, W. S. Shin, *Adv. Energy Mater.* **2019**, *9*, 1900168.
17. H. S. Kim, S. Rasool, W. S. Shin, C. E. Song, D. H. Hwang, *ACS Appl. Mater. Interfaces* **2020**, *12*, 50638.
18. T. Lee, S. Oh, S. Rasool, C. E. Song, D. Kim, S. K. Lee, W. S. Shin, E. Lim, *J. Mater. Chem. A* **2020**, *8*, 10318.
19. H. S. Kim, C. E. Song, J. W. Ha, S. Lee, S. Rasool, H. K. Lee, W. S. Shin, D. H. Hwang, *ACS Appl. Mater. Interfaces* **2019**, *11*, 47121.
20. K. Zhang, Z. Chen, A. Armin, S. Dong, R. Xia, H. L. Yip, S. Shoaee, F. Huang, Y. Cao, *Sol. RRL* **2018**, *2*, 1700169.
21. S. Dong, K. Zhang, X. Liu, Q. Yin, H. L. Yip, F. Huang, Y. Cao, *Sci. China Chem.* **2019**, *62*, 67.
22. S. Rasool, J. W. Kim, H. W. Cho, Y. J. Kim, D. C. Lee, C. B. Park, W. Lee, O. H. Kwon, S. Cho, J. Y. Kim, *Adv. Energy Mater.* **2023**, *13*, 2203452.
23. S. L. Lim, K. H. Ong, J. Li, L. Yang, Y. F. Chang, H. F. Meng, X. Wang, Z. K. Chen, *Org. Electron.* **2017**, *43*, 55.
24. D. I. Kutsarov, E. New, F. Bausi, A. Zoladek-Lemanczyk, F. A. Castro, S. R. P. Silva, *Sol. Energy Mater. Sol. Cells* **2017**, *161*, 388.
25. Y. M. Chang, C. Y. Liao, C. C. Lee, S. Y. Lin, N. W. Teng, P. Huei-Shuan Tan, *Sol. Energy Mater. Sol. Cells* **2019**, 202, 110064.
26. J. Zhang, Y. Zhao, J. Fang, L. Yuan, B. Xia, G. Wang, Z. Wang, Y. Zhang, W. Ma, W. Yan, W. Su, Z. Wei, *Small* **2017**, *13*, 1700388.
27. H. Jung, J. Kim, J. Park, M. Jahankhan, Y. Hwang, B. Kang, H. Kim, H. Y. Park, P. Ahn, D. H. Um, J. S. Jee, W. S. Shin, B. S. Kim, S. H. Jin, C. E. Song, Y. Lee, *EcoMat* **2024**, *6*, e12421.
28. Z. U. Rehman, M. Haris, S. U. Ryu, M. Jahankhan, C. E. Song, H. K. Lee, S. K. Lee, W. S. Shin, T. Park, J. C. Lee, *Adv. Sci.* **2023**, *10*, 2302376.
29. C. Wang, X. Ma, Y. fan Shen, D. Deng, H. Zhang, T. Wang, J. Zhang, J. Li, R. Wang, L. Zhang, Q. Cheng, Z. Zhang, H. Zhou, C. Tian, Z. Wei, *Joule* **2023**, *7*, 2386.
